# Computer Simulations of Deep Eutectic Solvents: Challenges, Solutions, and Perspectives

**DOI:** 10.3390/ijms23020645

**Published:** 2022-01-07

**Authors:** Dmitry Tolmachev, Natalia Lukasheva, Ruslan Ramazanov, Victor Nazarychev, Natalia Borzdun, Igor Volgin, Maria Andreeva, Artyom Glova, Sofia Melnikova, Alexey Dobrovskiy, Steven A. Silber, Sergey Larin, Rafael Maglia de Souza, Mauro Carlos Costa Ribeiro, Sergey Lyulin, Mikko Karttunen

**Affiliations:** 1 Institute of Macromolecular Compounds, Russian Academy of Sciences, Bolshoy pr. 31, 199004 St. Petersburg, Russia; luk@imc.macro.ru (N.L.); kubastyi@gmail.com (R.R.); nazarychev@imc.macro.ru (V.N.); natalia.borzdun@gmail.com (N.B.); i.v.volgin@gmail.com (I.V.); andreeva.masha@mail.ru (M.A.); glova.temik@gmail.com (A.G.); sofia.melnikova122@gmail.com (S.M.); aleksey_dobrovsky@mail.ru (A.D.); selarin@macro.ru (S.L.); s.v.lyulin@gmail.com (S.L.); 2 Department of Physics and Astronomy, The University of Western Ontario, 1151 Richmond Street, London, ON N6A 5B7, Canada; poseidon.prime@gmail.com; 3 The Centre of Advanced Materials and Biomaterials Research, The University of Western Ontario, 1151 Richmond Street, London, ON N6A 5B7, Canada; 4 Departamento de Química Fundamental, Instituto de Química, Universidade de São Paulo, Avenida Professor Lineu Prestes 748, São Paulo 05508-070, Brazil; rafael.maglia.souza@usp.br (R.M.d.S.); mccribei@iq.usp.br (M.C.C.R.); 5 Department of Chemistry, The University of Western Ontario, 1151 Richmond Street, London, ON N6A 5B7, Canada

**Keywords:** deep eutectic solvents, computer simulation, quantum mechanics, molecular dynamics, machine leaning

## Abstract

Deep eutectic solvents (DESs) are one of the most rapidly evolving types of solvents, appearing in a broad range of applications, such as nanotechnology, electrochemistry, biomass transformation, pharmaceuticals, membrane technology, biocomposite development, modern 3D-printing, and many others. The range of their applicability continues to expand, which demands the development of new DESs with improved properties. To do so requires an understanding of the fundamental relationship between the structure and properties of DESs. Computer simulation and machine learning techniques provide a fruitful approach as they can predict and reveal physical mechanisms and readily be linked to experiments. This review is devoted to the computational research of DESs and describes technical features of DES simulations and the corresponding perspectives on various DES applications. The aim is to demonstrate the current frontiers of computational research of DESs and discuss future perspectives.

## 1. Introduction

Over the past two decades, deep eutectic solvents (DESs) have become increasingly sought after for a wide range of applications [1,2]. According to Martins et al. [3], who contributed significantly to the formulation of the comprehensive definition of DES from a thermodynamic point of view, DESs are eutectic mixtures of two or more pure components with the eutectic point temperature significantly lower than that of an ideal liquid mixture. This was first discovered by Abbott et al. [4] using a mixture of choline chloride (ChCl) and urea (in the molar ratio 1:2), which is so-called reline, currently one of the most studied DESs. A mixture of ChCl and urea at a molar ratio of 1:2 melts at 30 °C [5] and the corresponding ideal solution melts at 100 °C.

Since the first publications in 2001 [4], the number of articles devoted to DESs has increased exponentially and to-date there are over six thousands publications on this topic, according to Web of Science. This great interest in DESs is due to their unique properties. DESs are often classified as cheap, non-toxic, and environmentally friendly analogues of ionic liquids (ILs); see, e.g., Amde, Liu, and Pang [6] for a review regarding environmental aspects of ILs. DESs have indeed shown great potential for nanotechnology [7], electrochemistry [8], extraction processes [9], biomass transformation [10], additive technology [11,12], pharmaceuticals [13,14], biosensor development [15], membrane technology [16,17], and many other applications. 

Despite the fact that DESs have similar physical properties to ILs, they differ significantly in their chemical structures. DESs are mixtures of at least two compounds that have different types of interactions, which are the underlying reason for the strikingly deep melting points.

DESs are typically classified into four types depending on their chemical nature, as listed in Table 1. Type III deserves special attention since it includes a subclass of natural DESs (NADESs). NADESs are special in the sense that their components are derived from natural resources. The term “natural DES” was first proposed by Choi et al., 2011 [18]. NADESs are biocompatible and biodegradable which expands their potential applications to the medical field. The extensive growth in DES development has led to the appearance of new types of DESs. Thus, in 2018 Verma et al. [19] discovered a deep depression in the melting temperature of the mixture of non-ionic compounds (menthol and organic acids). Subsequently, Abranches et al. [20] also discovered this effect in a mixture of thymol and menthol and suggested classifying it as a new type of DES-non-ionic DES. This discovery expands the properties and possible applications of DESs and has instilled a lot of interest from both experimental and computational researchers. It is important to point out that DESs are multi-component systems and consist of various types of compounds (salts, acids, alcohol, etc.). The versatility of the components opens up broad opportunities for DES development with highly tuneable properties. 

**Figure 1:**
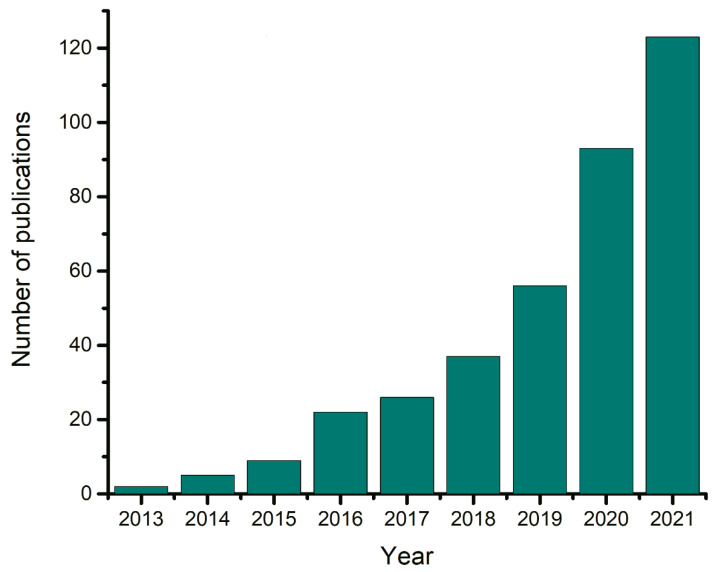
Number of publications per year with keywords “DESs + simulation” in the Web of Science.

One of the proven approaches to targeted material development is combined experimental and theoretical research [21,22]. Theory is used to determine the relationship between the structure and properties and can serve as a guide for further experiments. Computer simulations have their own methodological and practical issues, but they are unique in that they allow for direct observations of intermolecular interactions and processes at the nanoscale [23,24]. According to Web of Science, the number of studies devoted to computer simulations of DESs has been increasing exponentially since 2013 (Figure 1). In addition, the development of artificial intelligence (AI)-based approaches offers a new way to establish links between the structure and material properties.

Computer simulations that are directly related to DESs can be divided into different groups. Most of the works are devoted to the structure and dynamical properties of DESs to understand how those relate to the composition. Another important direction is low molecular weight compounds in DESs with a main focus on the development of separation techniques for CO_2_ and SO_2_ removal, as well as biofuel purification [9,25,26]. In nanotechnology, DESs are applied as solvents for the development of nanoparticles [7]. In this context, knowledge about the interactions of the nanoparticle surfaces with DES molecules, and their influence on DES structure and dynamics, are fruitful targets for simulations. Since DESs are used as electrolytes in power systems and battery technology [27,28,29], interactions of DESs with electrode surfaces is an important subject. Recent studies have also suggested the potential of DESs as storage media for biomolecules [30], and even as solvents for drugs [31] and pharmaceutical agents [32,33]. In all of the above cases, it is extremely important to understand how DESs affect the molecular conformations, which can be revealed by computer simulation methods, such as atomistic molecular dynamics (MD) simulations. The last but not least popular DES application is biomass formation, where a DES serves as a solvent for carbohydrates. In this case, simulation techniques can be used to reveal the molecular mechanisms and interactions responsible for the solvation of carbohydrates. The most important area of study is perhaps the influence of water molecules on the properties of DESs. Since in practice it is almost impossible to remove water from DESs, and the water molecules dramatically affect the H-bonding network in it [34,35,36], understanding the influence of water is of critical importance.

In this review, we focus on simulations of DESs. Currently, there are only two reviews in which simulations of DESs are the central topic: Kovács et al. [37] reviewed the modeling of NADESs and Alkhatib et al. [38] devoted much attention to simulations of DESs in their review on thermodynamic modeling of DESs. These reviews were published at the end of 2019. Since then, the number of articles devoted to simulations of DESs has almost doubled (Figure 1). Some computational studies have been discussed in reviews devoted to current DES applications. For example, Kaur et al. [39] reviewed the research on microstructure and devoted part of the review to simulations of DES structures. Pelaquim et al. [17], Liu et al. [16], and Shama et al. [40] have provided overviews of studies devoted to modeling gas solubility in DESs. De Castilla et al. [41] discussed research on simulating thermodynamic and transport properties of DESs in their review. Ma et al. [35] analyzed the effect of water on DESs and provided a review of related simulations.

In this article, we review the status of computer simulations devoted to DESs. We discuss simulations of DESs at different scales (quantum chemistry calculations, atomistic modeling, and coarse-grained simulations) and also address machine learning techniques. It is important to note that there are also other computational methods that have been used for establishing links between material properties and structure. Popular methods for DES investigations include thermodynamic modeling, such as Conductor like Screening Model for Realistic Solvents (COSMO-RS) [42,43] and Statistical-Associating Fluid Theory (SAFT) equation of state [44,45,46,47] and its variants. COSMO-RS was developed by Andreas Klamt in 1995 and is now a very popular method for investigations of liquid properties. Based on quantum mechanical calculations of the charge densities of the molecules and estimation of interactions between the different segments of the liquid compounds, this method allows for the prediction of chemical potentials and properties of DES compounds without resource-intensive calculations. It gives a unique opportunity to screen large amounts of DES compounds for developing DESs with desired properties. In addition, this technique can be used for validations of MD simulations [48]. SAFT [44,45,46,47] is a method based on the determination of the residual Helmholtz energy as the sum of a reference term and terms for the molecular interactions and associations. A variation of this method was first used for the investigation of DES solubility in 2015 [49] and it has become a common method for DES development. These methods are being actively developed and, for example, could be combined to increase predictive power [50]. In our review, we focus on molecular modeling and AI techniques. For more detailed discussions of thermodynamic models in DES research, we refer to the recent reviews [38,41,51]. 

## 2. Simulation Methods for DESs

### 2.1. Quantum Mechanical Methods

Interactions between molecules in DESs are more diverse than in regular liquids and understanding them is more difficult. Namely, the nature of the H-bond network and charge delocalization between solvent components are some of the key matters of interest. The first general consideration about the physical mechanisms in reline was the suggestion that when urea interacts with chloride anions, they disrupt the choline chloride’s lattice, which leads to charge delocalization and prevents crystallization [52]. Later, it was shown by Altamash et al. using electronic structure calculations that the greatest charge transfer occurs from the anion to the choline cation [53]. Matters are more complex, however, and it has also been shown that the interactions between urea and chloride do not necessarily lead to a decrease in the melting point [54,55]. 

#### 2.1.1. DFT-Derived Peculiarities of the Local DES Structure

Since electronic structure methods are very demanding when it comes to computational resources, it is necessary to select a small but sufficient fragment of a DES for the simulation. As a good compromise between accuracy and efficiency at the quantum level, density functional theory (DFT) is typically the preferred method as it can provide a good description of geometry and electronic structure with reasonable calculation times. For example, DFT functionals are commonly used to calculate equilibrium geometries of single molecules or complexes of molecules bound by networks of various interactions in the gas phase or by applying continuum solvation models [56] to simulate the effects of a solution [56,57,58]. 

The conformational space of mutual arrangements of molecules in a DES can be studied using, e.g., semi-empirical methods (SE), such as the PM6 approximation [59,60] or the SE tight-binding DFT method GFN2-xTB [61]. DFT can also be used to study the details of the type and intensity of the interactions, as well as to quantify the short-range interactions. Methods to study such properties include Bader’s quantum theory of atoms in a molecule (QTAIM) [62], electrostatic potentials (ESP) and reduced density gradients (RDG). Classification of H-bonds, the corresponding bond strengths and covalency can be completed by analyzing bond critical points (BCP) in the QTAIM representation (as an example of a simple system, see Ref. [63]). Based on electron density and its derivatives, RDG analysis can characterize non-covalent interactions such as H-bonds, van der Waals interactions, and steric effects [64].

As for functionals, a lot of studies of systems containing no more than several dozen atoms have been performed using the hybrid B3LYP functional with 20% HF exchange in conjunction with the family of Pople’s split-valence basis sets with the addition of a different number of diffuse and polarization functions (6-31G(d), 6-31G(d,p), 6-31+G(d,p)) [61,64,65,66,67,68,69,70,71,72,73,74], and correlation-consistent basis sets with augmented diffuse functions aug-cc-PVDZ [75]. Some organic cations contain alkyl side chains and/or aromatic moieties with important contributions from dispersion forces and hence require a proper description. This can be completed by amending the density functionals with dispersion corrections, such as Grimme’s D2 [72,76], D3 [64,77,78,79], and D3BJ [80]. Another popular alternative is to use the meta-hybrid functional M06-2X with 54% HF exchange. It has been shown to have excellent performance and accuracy in systems where dispersion interactions contribute significantly to conformer energetics [67,81,82,83,84,85,86,87]. Other functionals, for example hybrid long-range corrected CAM-B3LYP [67], hybrid PBE0 [67], hybrid PW91 [88] have also been used, albeit rarely.

DFT approaches have not only been fruitful for studies of distribution patterns of DES components, but also in studies of interactions of DESs with various functional substances in gas separation. In particular, free energy changes and structural analyses have been used to develop new solutions towards desulfurization of liquid fuels [73,78,85,86,89], capturing greenhouse gases such as CO_2_ or SO_2_ [72,79,90,91], metronidazole extraction from plasma [92], developing efficient mercury removal strategies from different gases [93], extractive detoxification of feedstocks for the production of biofuels using new hydrophobic DESs [74], capturing NH_3_ [94], and for separating phenolic compounds from oil mixtures [95].

Even more complex problems have been investigated in the context of nano-objects. Lawal et al. [96] provided a molecular-level description of the interactions controlling a DES composed of a mixture of methyltriphenylphosphonium bromide with glycerol and carbon nanotubes and revealed physisorption through hydrophobic and π–π interactions. Shakourian-Fard et al. [87] used the M06-2X functional to analyze the electronic structure of noble metal nanoparticles (M_n_, M = Cu, Ag, and Au; *n* = 1–4) and their complexes with ChCl:Urea DES. The study revealed two major bonding factors that govern the interactions: the [Cl]^–^…Mn interaction and unconventional H-bonds (C–H...M_n_ and N–H...M_n_). Shakourian-Fard et al. [87] used the M06-2X/cc-pVDZ level to characterize adsorption of DESs on different graphene surfaces and showed that it is non-covalent and dominated by dispersion energies.

#### 2.1.2. Relations between DFT and NMR and FTIR Experiments of DESs

It is imperative to compare computational predictions with experiments, for example, Fourier-transform infrared spectroscopy (FTIR) or nuclear magnetic resonance (NMR). NMR enables studies of structure-property relationships and interactions in DESs by probing both cations and anions through several nuclei (^1^H, ^13^C, ^19^F, ^35^Cl, ^11^B, ^15^N, and ^31^P) [97]. The use of NMR chemical shift deviations, relaxation, nuclear Overhauser effect, and diffusion experiments allows for advanced studies of interactions between cation, anion and solute, and, consequently, facilitates the molecular design of DESs. Li et al. [89] used the B3LYP/6-311+G(2d,p) level with the self-consistent reaction field solvation model to study the solvation effects of dimethylsulfoxide (DMSO). They calculated the NMR shielding constants of hydrogen atoms by the gauge-including atomic orbitals (GIAO) method [98] for different SO_2_^−^ anion adducts of DESs and revealed molecular details of sorption.

FTIR is a reliable technique for analyzing not only liquids but also solid samples. Vibrational modes from DFT calculations may be matched against FTIR absorption spectra. The presence of hydrogen bonds can be deduced from FTIR spectra after vibration assignments. The accuracy is determined by the system size and how well the range of internal vibrational frequencies inherent in the system is covered. As an example, Araujo et al. [70] used a combination of computational and vibrational spectroscopy tools, including inelastic neutron scattering (INS), to probe intermolecular interactions in a eutectic mixture of reline. Their analyses showed that reaching an agreement between calculations and experiments requires expanding the models to include a greater variety of molecular contacts. They performed comparative calculations of a discrete model of a single cluster by utilizing the B3LYP functional and calculations of a periodic model of the aggregate by using the plane-wave pseudopotential method with the Perdew–Burke–Ernzerhof (PBE) exchange-correlation functional [99]. The eigenvalues and eigenvectors from the Gaussian’s frequency calculation of a single cluster were then used to estimate the intensities of the INS spectrum. The phonon frequencies for the aggregate were obtained by diagonalization of the dynamical matrices computed using density-functional perturbation theory [100]. As a result, the region above 200 cm^−1^ in reline’s INS spectrum is satisfactorily described by the discrete cluster approximation. The simulations, however, failed in the lowest frequency region, which, as mentioned by the authors, may only be adequately represented by a three-dimensional lattice.

The phonon modes of the “shock-frozen” reline in an amorphous phase representing a 3D array of extended clusters also did not perfectly succeed in replicating reline’s low-frequency modes. Araujo et al. [70] noted that while discrete ab initio calculations of internal vibrational modes satisfactory match to INS experiments, a little improvement is achieved by running periodic calculations of the aggregate. The authors also noted that the advantage of the periodic over the cheaper discrete calculation is a better representation of the pure solvent components, whose details of crystal lattice packing is impossible to reproduce using a small cluster. Hence, when going from pure solvent components to their eutectic mixture, it requires a certain model of the pure crystal lattices for a realistic comparison of calculated and experimental frequency shifts.

#### 2.1.3. Periodic DFT in Studies of the Condensed Phase of DESs

Periodic ab initio calculations have become a popular tool allowing the study of hundreds of atoms. This method serves as a powerful instrument to investigate the electronic structure of the condensed phase of DES, but the system sizes are still limited. Usually, periodic ab initio calculations use the DFT in the hybrid Gaussian and plane waves (GPW) approach [101].

Korotkevich et al. [102] used this approach to study SO_2_ absorption by ChCl/glycerol DES. The molecularly optimized double-z basis set (MOLOPT-DZVP-SR-GTH) [103] was applied to all atoms together with the generalized gradient approximation (GGA) utilizing the Becke–Lee–Yang–Parr (BLYP) [104,105] functional and the corresponding BLYP Goedecker–Teter–Hutter (GTH) [106] pseudopotentials for core electrons. The deficiency of the dispersion interactions for the GGA functional was corrected using Grimme’s D3 scheme with Becke–Johnson damping [107,108]. The authors identified hydrogen bonding and other specific interactions between all components. Fetisov et al. [109] used the same approach to conduct ab initio MD (AIMD) simulations in the canonical ensemble at temperatures of 333 and 363 K to investigate the behavior of reline and its equimolar mixture with water. It was shown that in hydrous reline, water competes for the anions, and the hydrogen atoms of urea have similar propensities to bond to the chloride (Cl^−^) ions and the oxygen atoms of urea and water. The same level of theory was used by Malik et al. [90] to elucidate the solvation structure around CO_2_ and SO_2_ in ChCl-based DESs, namely, reline and ethaline. Zahn et al. [110] revealed significantly reduced ion charges in several choline-based DESs by using ab initio molecular dynamics in the GPW representation.

### 2.2. Molecular Dynamics Simulations

MD simulations have become the most popular computational technique for studies of nanostructures and dynamic properties of materials [111]. MD provides direct information about the molecular processes, and can explain and predict molecular interaction mechanisms. More than half of the computational papers devoted to studies of DESs (more than 150 articles in the Web of Science by the end of 2021) use MD simulations. Most commonly, MD simulations are used to obtain information about the nanoscale structure. Another frequent goal is to study properties directly related to applications, such as gas separation and fuel purification, i.e., simulations of low molecular weight compounds in DESs. The accessible length scales, typically on the order of about 10 nm, allow simulations of nano-sized objects and surfaces of larger objects. Importantly, MD simulations are also a useful tool for investigations of rheological properties. The MD technique has, however, its own limitations, see, e.g., Refs. [112,113,114].

The main challenge in MD simulations is the development of force fields (FFs) capable of reproducing the structural and dynamic properties of DESs. The history of FF development for DESs is inextricably linked to the development of FFs for ionic liquids, since the interactions in these solutions are very similar. However, due to the differences in compositions and specific interactions in them, the FFs for DESs have their own peculiarities. The main problems are related to the presence of strong ionic interactions and highly polarizable atoms and molecules, and thus neglecting polarization may lead to an overestimation of the ion–ion interactions [115] and potentially unreliable results, such as a reduction in the diffusion coefficient by several orders of magnitude [115]. However, non-polarizable FFs are often able to correctly reproduce the structural properties of DESs [116].

There are two main directions currently being pursued to solve the problem regarding polarization effects. The first is based on adding correction(s) to the non-covalent interactions in existing FFs. This approach has the advantage that it uses existing FFs and no additional parameterization is needed. Moreover, this approach does not require more computational resources than the usual MD. As a result, this has been the most common approach used in simulations of ionic liquids [117]. The second approach is the explicit inclusion of polarization effects in force fields, based on formulations that model the electronic degrees of freedom and thus requires more computational resources. This second approach is described in the next section.

The first approach is based on charge rescaling. Since the atomic charges in DESs have a significant impact on macroscopic properties [118], rescaling of the electrostatic interactions is an effective way to reparametrize FFs for DESs. The magnitude of rescaling has varied from 0.78 to 0.9 (e.g., Refs. [119,120,121]). Rescaling charges helps to reduce overbinding and achieve agreement with experimental values regarding dynamic properties [122]. However, this approach has its drawbacks. Since the magnitude of rescaling could depend on particular details of the system, a change in composition should be accompanied by new validation of the FF. A common approach for FF validation is to select the scaling parameter such that it reproduces experimental data, usually density, viscosity, and diffusion coefficients (e.g., Refs. [123,124]). Although charge scaling helps, at least in some cases, to achieve agreement with experiments, it can also lead to artificial structural and dielectric properties, such as an excessive decrease in density [125], less intense peaks in the radial distribution functions, and an artificial dielectric response [126]. In addition, changes in the atomic charges can affect the parameterization of intermolecular interactions leading to artificial structural characteristics [122,127]. One particular problem is the incapacity of the scaled-charge models to fix the artificially enhanced long-range ion–ion correlations present in non-polarizable models, as discussed by McDaniel and Yethiraj [128]. In addition to that, Son et al. [129] have shown that mixtures of compounds are not well reproduced with scaled-charge models, since these models underestimate the cohesive energy and lead to poor predictions of phase behavior.

As the above indicates, finding new approaches to develop transferable FFs is of critical importance. For example, Chaumont et al. [130] proposed a reparameterization of the van der Waals potentials for atoms involved in H-bonding as an alternative to charge rescaling. Another fruitful approach is explicit inclusion of polarization. Compared to fixed-charge FFs, polarizable FFs are significantly more demanding on computational resources but are more accurate and help to reproduce both structural and dynamic properties. Polarizable FFs are discussed in detail in the next section. The main advantage of fixed-charge FFs is their high transferability which allows for easy changes of DES compositions and simulations of diverse compounds. However, despite their ability to model and predict structural characteristics, their lack of accounting of polarization is a major drawback. Corrections to existing fixed charge FFs allow to achieve correct results on dynamic properties, but that often comes at the expense of transferability and can lead to unexpected artificial results.

### 2.3. Polarization and Polarizable Force Fields for Deep Eutectic Solvents

There are several ways to introduce polarization effects such as fluctuating charges [131,132], induced point dipoles [133], or Drude oscillators [134,135]. Fluctuating charges allow mimicking polarizability with a, respectively, low computational cost. However, in some cases, it might overestimate the polarizability of the molecule [136] and cannot simulate induction of the out-of-plane dipole moment in planar molecules. The induced point and Drude-induced dipoles are based on using additional particles, either as massless points (induced point dipoles) or as positive and negative charges connected to the atoms by a spring. Schmollngruber et al. have shown that there are no quantitative differences between these two methods in simulations of molecular IL of 1-ethyl-3-Methyl-imidazolium triflate [137].

The high concentration of ions in DESs results in non-negligible local electric fields that polarize the components of the eutectic solution. So far, almost all MD simulations of DES have been performed with non-polarizable models only. However, efforts are emerging towards simulations with polarizable FFs [138,139,140,141]. They are all based on the classical Drude oscillator model, which will be briefly described next. For a deeper understanding of this method, as well as other polarization methods, see the review of Bedrov et al. [115].

The Drude oscillator model consists of adding an additional particle, the Drude particle (DP), bonded to the nucleus, also called the Drude core (DC), by a harmonic potential. In this arrangement, the DP receives a negative charge (−*q_D_*), while the DC receives a positive charge (*q_D_*), which is summed onto the initial charge of that nuclei, forming a Drude induced dipole. This dipole is intended to capture the distortion of the electron cloud, since the DPs are free to move around the nucleus. The pair of charges (±*q_D_*) assigned to the DP and DC of a polarizable atom are determined by the atomic polarizability of that atom and the force constant of the harmonic potential that connects the DP to the DC. In addition to that, the polarizability of the hydrogen atoms is usually added onto the polarizabilities of the heavy atoms that own them; hydrogens themselves are treated as non-polarizable because of their small atomic mass. The relation between the polarizability (α), force constant (*k_D_*), and Drude charges (±*q_D_*) is given by

(1) $$\alpha =\frac{q_{D}^{2}}{k_{D}}$$

#### 2.3.1. The Polarizable CL&Pol Force Field

In addition to the high computational cost, another main problem with polarizable FFs is their poor transferability. Recently, using the CL&P fixed-charge FF [142] as the basis, Goloviznina et al. developed a transferable polarizable FF for ionic liquids [143] and extended it to DESs [139,142]. In order to increase transferability, they applied a fragment approach, which was validated by calculating density, ion diffusion coefficients, and viscosity for a range of ionic liquids and their mixtures [143]. In DESs, the strong combination of H-bonds and Drude-induced dipoles causes stability problems which were also addressed by Goloviznina et al. [139].

When converting the non-polarizable CL&P model to the polarizable CL&Pol, the first step is the addition of the Drude induced dipoles in the heavy atoms based on Equation (1). Atomic polarizabilities are determined by first principle calculations. In CL&Pol [138,139], the force constants of all of the harmonic bonds between the DCs and the DPs are assigned to be *k_D_* = 4184 kJ∙mol^−1^ and the masses of all DPs to m_DP_ = 0.4 u. It is also necessary to scale down the Lennard–Jones energy parameter 𝜀 to avoid double counting the polarization effects, since induction effects are implicitly included in the CL&P parameter set [142].

In principle, this scaling can be rigorously performed based on symmetry-adapted perturbation theory [144] (SAPT) calculations, a quantum chemistry method that allows decomposing the interaction energies into electrostatic, induction, dispersion, and repulsive terms. However, SAPT calculations are computationally intensive, especially if applied to a broad range of compounds. In this context, an alternative predictive scheme was devised by Goloviznina et al. [138,139,143] to obtain the scaling factor (*k_ij_*) for the interaction between fragments *i* and *j*,

(2) $$k_{ij}={\left(1+c_{0}r_{ij}^{2}\frac{Q_{i}^{2}\alpha_{j}+Q_{j}^{2}\alpha_{i}}{\alpha_{i}\alpha_{j}}+c_{i}\frac{Q_{i}^{2}\mu_{j}+Q_{j}^{2}\mu_{i}}{\alpha_{i}\alpha_{j}}\right)}^{-1}$$

This scheme only needs basic molecular properties of the target fragments: net charges (*Q*) dipole moments (*μ*), and polarizabilities (*α*). In addition, *r_ij_* is the equilibrium distance of the fragments, and $c_{0}=0.25$ and $c_{1}=0.11$ are coefficients that were adjusted to a set of reference *k_ij_* values, obtained from SAPT calculations that covered charged and neutral key-fragments. These key-fragments are common molecular structures present in a broad range of DESs components, which allow to achieve a good transferability. For instance, triethylammonium cations are represented by trimethylammonium and butane as fragments.

All of the intramolecular bonded parameters and initial atomic partial charge distributions are simply taken from the CL&P FF [142]. In addition, for modeling DESs with CL&Pol, two new potentials are present. These are the Tang–Toennies [145] and Thole [146] damping functions,

(3) $$f_{n}\left(B_{ij},r_{ij}\right)=1-ce^{-B_{ij}r_{ij}}\sum_{k=0}^{n}\frac{{\left(B_{ij}r_{ij}\right)}^{k}}{k!},$$

(4) Trij=11+prij2αiαj16e−prij/αiαj16,

where *k* = 4 is the order of the sum, *B* = 4.5 determines the spatial extension of the damping, *c* = 1, *r_ij_* is the distance between the sites, *i* is the polarizability of atom *i*, and *p* = 2.6 is the Thole Parameter.

The Thole function is used to dampen, at short distances, the Coulomb interactions originating from the induced dipoles. The Tang–Toennis function dampens short-range charge–dipole interactions, avoiding instabilities in the MD simulations. These functions avoid the “polarization catastrophe” [115,139], that is, excessive correlations between nearby dipoles and also events in which the DPs are captured by neighboring DCs.

An important aspect when using the CL&Pol FF is the absence of Lennard–Jones parameters in some of the hydrogen atoms, especially those involved in H-bonds, such as the hydroxyls group of choline cations and polyol molecules. These hydrogen atoms are referred to as “naked” hydrogens [138,139]. This particular aspect may lead to unrealistically strong interactions after the addition of the Drude particles. This can be circumvented by increasing the atomic diameter (𝜎-parameter) of the heavy atom to which, such as a hydrogen, is bound [138,139]. Recently, de Souza et al. [147] showed that using the values of the 𝜎-parameter of the CL&Pol FF leads to an artificial phase separation between the components of the DES ethaline. This can be fixed by carefully adjusting the 𝜎-values of the hydroxyl groups to reproduce ab initio radial distribution functions.

There is emerging evidence about the so-called “chloride’s overpolarization” that may be present in polarizable MD simulations. This was first noted by Szabadi et al. [147], who performed aqueous chloride-based ionic liquid simulations, and subsequently reported by de Souza et al. [140] in DES ethaline. This issue has its physical origin in the high polarizability of chloride, Cl = 4.4 Å^3^. In the work of Szabadi et al. [147], they noted an artificial alignment of chlorides with water molecules. In turn, de Souza [140] found that this overpolarization leads to an overestimated spatial nano-heterogeneity, as indicated by peaks and anti-peaks at very low q-vector values in all self- and cross-correlations of the partial X-ray structure factors. Szabadi et al. [147] tried to reduce the chloride’s polarizability, while de Souza et al. [140] extended the application of the Tang–Toennis damping function for chloride’s induced dipole interactions. In both cases, the behavior of the polarizable MD simulations improved.

The aforementioned aspects of the CL&Pol FF (“naked” hydrogens and chloride’s overpolarization) may make it difficult to apply in simulations of DESs. Nonetheless, following the guidelines of the original CL&Pol FF [138,139] and the contributions from de Souza et al. [140] facilitate its reliable use. In addition, the CL&Pol FF has parameters available for a broad range of components of DESs, such as alkylammonium-based and alkylphosphonium-based cations, urea, ethylene glycol, and so on. In addition to that, the CL&Pol model is relatively easy to extend to other components, since it shares the functional form and parameterization strategy of the OPLS FF.

#### 2.3.2. The Polarizable SAPT Force Field

The second available polarizable FF for DESs is the model from Jeong et al. [141], which we will refer to as SAPT-FF since it is completely obtained from scratch based on a SAPT protocol [148]. In this approach, the atomic point charges are obtained using a distributed multipole analysis (DMA) on the electron density of a single molecule. Then, the intermolecular parameters from distinguished FF terms are individually fitted to the components of the total non-bonded energy, composed of electrostatics, induction, exchange, dispersion, and delta Hartree–Fock, all obtained from SAPT calculations. The functional form of these energy components are given as

(5) $$E_{elec}\approx \sum_{ij}\frac{q_{i}q_{j}}{r_{ij}q_{j}}+\sum_{ij}A_{ij}^{elec}e^{\left(-B_{ij}r_{ij}\right)},$$

(6) $$E_{pol}\approx U_{shell}^{\left(2\right)}+\sum_{ij}A_{ij}^{ind}e^{\left(-B_{ij}r_{ij}\right)},$$

(7) $$E_{exch}\approx \sum_{ij}A_{ij}^{exch}e^{\left(-B_{ij}r_{ij}\right)},$$

(8) $$E_{disp}\approx \sum_{n=6,8,10,12}\sum_{i,j}f_{n}\left(B_{ij},r_{ij}\right)\frac{C_{n}^{ij}}{r_{ij}^{n}},$$

(9) $$E_{pol}\approx U_{shell}^{\left(\Delta \mathrm{SCF}\right)}+\sum_{ij}A_{ij}^{\delta hf}e^{\left(-B_{ij}r_{ij}\right)}.$$

The electrostatic energy ($E_{elec}$) is composed of a Coulomb potential and a short-range term describing charge penetration effects. The induction energy ($E_{pol}$) is the contribution from the polarizable Drude oscillator (the second-order Drude oscillator energy) plus a short-range charge penetration component. The exchange energy ($E_{exch}$) describes the short-range repulsion with an exponential Born–Mayer function. The dispersion energy ($E_{disp}$) is represented with a series of r–n power functions (*n* = 6, 8, 10, 12) and it is dampened by $f_{n},B_{ij},r_{ij}$, the Tang–Toennis function of Equation (3). The delta Hartree–Fock energy is composed of the Drude oscillator energy above second-order and the penetration component of the 𝛿 Hartree–Fock energy. Furthermore, for each pair of atoms, the pre-exponential coefficients $A_{ij}^{elec}$, $A_{ij}^{ind}$, $A_{ij}^{exch}$, and $A_{ij}^{\delta hf}$ determined based on the SAPT energy benchmark. In addition, the exponents $B_{ij}\;$and the dispersion coefficients $C_{n}^{ij}$ are assigned with specific mixing rules [141].

The sum of all terms yields the total non-bonded energy expression,

(10) $$E_{total}\approx \underset{ij}{\sum }\frac{q_{i}q_{j}}{r_{ij}q_{j}}+U_{shell}+\underset{ij}{\sum }\left(A_{ij}^{tot}e^{\left(-B_{ij}r_{ij}\right)}-\underset{n=6,8,10,12}{\sum }f_{n}\left(B_{ij},r_{ij}\right)\frac{C_{n}^{ij}}{r_{ij}^{n}}\right),$$

where $U_{shell}=U_{shell}^{\left(2\right)}+U_{shell}^{\left(\Delta \mathrm{SCF}\right)}$ is the total Drude oscillator polarization energy and it considers all the intramolecular DP–DP interactions. Those atom pairs at 1–4 or closer distances are screened using the Thole function of Equation (4) with *p* = 2.0.

The costly case-by-case SAPT-based parameterization combined with the incompatibility of the specific functional form of the SAPT-FF with commonly used FFs are challenging aspects to achieving transferability. In fact, reline is the only available DES within the SAPT-FF [141]. However, this “physically motivated” SAPT-FF presents some advantages. In principle, any DES can be simulated in this formalism, considering that the FF is fitted to ab initio data and no prior experimental data are needed in advance. Furthermore, due to the explicit separation of energy components, improvements in the quality of individual parameters are possible without the need for complete reparameterization. In addition, the molecular interactions present in MD simulations of any DESs can be accurately rationalized in terms of the different energy contributions.

### 2.4. Combinations of Quantum Mechanics and Molecular Dynamics Techniques

The previous sections highlighted the advantages and disadvantages of different computational approaches to studying DESs. One way to unite the strengths of these methods and overcome their weaknesses is to use them in combination. In this regard, there are two fundamentally different approaches. The first one implies the simultaneous application of MD and QM methods within a single study. The second approach involves the use of advanced ab initio MD (AIMD) simulations, also known as first-principles MD. As quantum effects are not directly included in classical MD, several characteristics of the systems can be probed only with the use of QM approaches. Conversely, due to the complexity of QM calculations, the time and length scales accessible in classical MD are well beyond those available in QM.

Aparicio’s group has used a combination of QM and MD to investigate the properties of a number of DESs, including ones based on ChCl [52,53,54,55], ammonium [149], arginine [150,151,152], betaine [153], and cineole [154] for applications such as gas capture, drug delivery, oil desulfurization, and the development of task-specific solvents. In particular, the strength and localization of H-bonds, the binding energy of the ionic pairs, as well as energetically favored positions of solvated molecules with respect to DES molecules were obtained from DFT. Their MD simulations allowed the estimation of properties, such as intermolecular interaction energies, the extension of H-bonds, their number, residence times, as well as prediction of the physicochemical properties of the fluids.

The QM and MD methods are not always applied independently. For example, Ali et al. [155] used MD simulations to obtain the energetically most favorable cluster conformers of menthol-based DESs, which required sufficient simulation time, and then DFT calculations were utilized to optimize the isolated structures and calculate their structural and thermochemical properties. In a study on ChCl/acetylsalicylic acid (ASA) therapeutic DESs by Saha et al. [155], radial distribution functions obtained from MD simulations demonstrated the presence of several H-bonds between the components and DFT calculations allowed demonstrating that Cl^−^ is acting as a charge transfer bridge between choline and ASA. Finally, a combination of QM and MD methods is often not an independent research method, but an auxiliary way to confirm a hypothesis formulated on the basis of experimental findings, which is no exception in the case of DES studies [156,157].

Similarly to classical MD, in AIMD, Newton’s equations of motion are solved at each simulation step. However, instead of using a prescribed potential, DFT calculations are employed to calculate the energy, which is then considered a function of nuclear coordinates. Thus, AIMD directly treats many-body effects and polarizability. Moreover, compared to classical MD, AIMD does not struggle with the problem of poor transferability. The drawbacks of AIMD are small system sizes (~up to few 100 atoms) and short simulation times (picoseconds), as well as the need to apply corrections due to the lack of van der Waals interactions, similarly to DFT.

Although AIMD methods have long been used to study ILs, to date, only about a dozen papers are devoted to the studies of DESs utilizing this method. The first investigation of DESs by AIMD was reported by Zahn et al. in 2016 [110]. The authors examined several choline-based DESs, including the widely studied mixture of ChCl and urea. As the negative charge transferred from the halide anion to the organic compound was found to be negligible, the authors questioned whether the deep eutectic melting point is due to charge delocalization occurring through this hydrogen bonding.

Among the subsequent AIMD simulations of DESs, a number of papers have focused on gas capture [90,102,158]. Since many-body effects and polarizability are directly included in AIMD, it is excellent for elucidating the local solvation structure around the gas molecules. For example, Malik et al. [90] demonstrated for CO_2_ and SO_2_ in reline and ethaline that charge transfer between the solute and the chloride anion determines the shapes of the solvation shells, while the nature of the H-bond donor (HBD) is responsible for its organization around the solute. AIMD allows one to investigate the solvation of not only small gas molecules in DES, but also, for example, mercury solvation, as its capture represents a major challenge in natural gas processing [159]. In addition to structural factors, AIMD has also been used successfully to probe charge transfer and chemical reactions in DES, as shown by Carrasco-Busturia et al. [160] and Warrag et al. [159].

AIMD can also be applied to validate FFs for classical MD simulations [161]. For example, Jeong et al. [141] utilized AIMD to develop atomistic polarizable FF for reline. The authors reported that the resulting FF is in good agreement with both AIMD simulations and experimental data on the static structure factor and diffusion coefficient.

The final remark concerns the significant limitation of the AIMD method, namely its resource intensity. Typical simulation times are of the order of hundreds of picoseconds, while the available sizes of the systems reach only some hundreds of atoms [109]. For example, Carrasco-Busturia et al. [160] have studied the speciation and reactivity in AlCl_3_:urea DES using AIMD. Estimated time scales required for direct AIMD simulation of reactants, AlCl_3_ and urea, were found to be well beyond nanoseconds, which is not feasible using AIMD. To overcome this technical limitation, the authors devised an approach based on the simulations of the products they hypothesized to be observed in this DES, such as chloroaluminate anions and [AlCl_x_(urea)_y_] cations, which allowed them to determine the possible paths for aluminium electrodeposition in the battery anode with reasonable simulation times.

### 2.5. Coarse-Grained Models

Despite its strengths, all-atom MD is limited by its characteristic time and length scales and computational resources [162,163]. One of the possibilities to overcome the limitations imposed on the simulation times and system sizes is multiscale simulations. One of the critical steps in these schemes is the correct transfer of data between different levels of the representation. These data should contain both structural and thermodynamic properties.

Coarse-grained (CG) models are often built using structure reduction [162,164,165,166,167]. This requires the definition of a scheme to transform the atomic structure of the investigated system into a coarse-grained representation (mapping scheme) and the determination of the interaction parameters between individual types of CG particles, i.e., a FF.

Typically, each CG bead represents a group of atoms. Therefore, the transition from atomistic models to CG ones can significantly reduce the number of particles in a system and, consequently, the number of degrees of freedom but simultaneously may also lead to inaccuracies when computing properties via fluctuation-dissipation relations [168]. The other main disadvantage is the loss of essential chemical details. CG does, however, enable a significant expansion in system sizes and simulation times.

As in the case of all-atom MD, the choice of the FF is of critical importance. In general, the determination of interaction parameters is carried out individually for each calculated system, taking into account the specifics of the mapping scheme to convert an all-atom representation into a coarse-grained one [162,169,170,171,172].

It should be noted that one of the main drawbacks of CG models is the lack of general applicability (MARTINI [167] being the notable exception) since, as mentioned earlier, the choice of a mapping scheme and model parameters should be made taking into account the studied system and the problem that is being addressed. With an increase in the number of atoms included in each CG bead (i.e., with a decrease in the number of CG beads in a system), one obtains a greater gain in the speed and efficiency of modeling due to a decrease in accuracy. Another important problem in CG modeling is the issue of the transferability [173] of models and FF parameters between different thermodynamic states (for example, for modeling at different temperatures), as well as the relationship between the CG timescale and the actual timescale. Coarse-grained FFs from ILs [174] may offer alternatives that can be applied to DESs as well.

The well-established Dissipative Particle Dynamics (DPD) [175,176] method has been used in a few DES simulations. Hu et al. [177] and Fan et al. [178] used DPD to study the self-assembly process of the zeolitic imidazole framework (ZIF) based on sodium dodecyl sulfate and zinc nitrate complex with 2-methilimidazole in reline with water. The simulation results made it possible to describe the structure of the hierarchical porous structure of ZIF-8 and the mechanism of its formation. Fan et al. [179] used DPD to study microemulsions based on DES (ChCl/urea), tetrahydrofurfuryl alcohol (THFA), and diethyl adipate (DA). The simulations were used to calculate the phase diagram, which was found to be in good agreement with experimental data. The simulations also showed that the main driving force behind the formation of a microemulsion is the interaction between DA and amphiphilic THFA molecules associated with a change in surface tension. However, the main restriction of the DPD approach is related to the highly coarse-grained nature of the potentials, which enable only qualitative comparison with experimental data and all atom simulations.

Despite the limited amount of coarse-grained simulations, we expect an increase due to the new works devoted to the development of coarse-grained force fields. In particular, in the group of Marrink in Groningen, the popular MARTINI force field was first extended to IL models [180], and very recently they developed the first coarse-grained Martini model for type III DESs [181] capable of reproducing experimental data on density, structure, and thermal expansion, and has a good potential for transferability.

### 2.6. Machine Learning Methods

Designing new DESs usually relies on an intuitive understanding of the relationship between the chemical composition of a DES and its properties. This can make molecular design time-consuming and costly. Machine learning (ML) has become an increasingly popular approach to alleviate such problems. ML is based on statistical processing of large datasets and detecting correlations between input and output data (for example, between structure and property) and using them to predict properties of new compounds [1,182,183]. One of the most common applications of ML is prediction of the quantitative structure-property relationship (QSPR) [1,184]. For this purpose, the most frequently used mathematical models are Artificial Neural Networks (ANNs) and methods of regression analysis [1,182,184].

#### 2.6.1. Working Principles of ANNs

ANN models consist of a set of nodes connected to each other and distributed over several layers, similar to the neuron cells in a human brain [182]. The data analysis typically begins with translating the structure from a chemical to mathematical language by coding it with molecular descriptors, or features [184]. There are a number of different types of descriptors; they can originate, for example, from properties with a clear physical meaning (e.g., molecular mass, the number of carbons in the structure, HOMO or LUMO energies and other characteristics obtained through the quantum chemical calculations) or from any type of topological indices [185].

**Figure 2:**
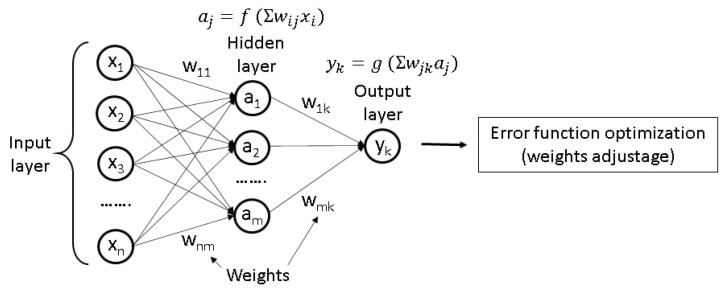
The scheme of a multilayer perceptron (a primitive model of ANN). Here, *w_ij_* and *w_jk_* are the connection weights, *f* and *g* are the activation functions.

After a translation of the structure to a set of its features has been performed, it can be regarded as an input layer of an ANN. The next step is transferring the initial data from one layer to another by applying a transformation function to the input data using appropriate weights at each node. The final layer consists of nodes that represent the properties of interest, for example, density or viscosity. As a result, the model can recognize some unknown non-linear correlations between the different features of the investigated material, although it does not explain the reasons for the existence of such correlations. In most cases, the ANN can be taught using training datasets, containing structures with already known properties. This training involves minimization of an error function and adjusting the weights [182]. A scheme of a simple commonly used model of ANN called “multilayer perceptron” is presented in Figure 2.

There are many methods to estimate the accuracy of the model, for example the mean absolute percent error, the relative error, the mean square error (MSE), the root mean square error, and so on. One of the most important and commonly used indicators of the model “goodness” is R^2^, defined as the ratio between the sum of squares regression and sum of squares total. In physical terms, R^2^ represents the proportion of dispersion in the dependent variable that can be explained by the independent variable, the closer its value to unity, the better the model describes the data.

#### 2.6.2. DES Property Prediction

There are many works dedicated to the prediction and estimation of the different properties of DESs by means of ANNs. For example, Shahbaz et al. used an ANN with three layers (6-9-1 architecture, namely the input, hidden, and output layer has 8, 4, and 1 neurons, respectively) to predict densities of three different ammonium- and phosphonium-based DESs across a range of temperatures and compositions [186]. The mole fraction of DES components and the temperature were used as inputs. They achieved an average absolute error of 0.14%. The same year, the authors published another study in which they applied an ANN model with an 8-4-1 structure for the prediction of glycerol removal from palm-oil-based biodiesel using DESs [187]. The results were in good agreement with the experimentally measured data with an absolute average deviation of 6.46%.

In another work by Benguerba et al., multi-linear regression (MLR) and ANN methods were utilized for the prediction of DESs’ viscosities [188]. The authors used the σ-profile surface area descriptors derived from DFT and a temperature descriptor as inputs for their ML models and 10^8^ experimental measurements of five amine-based DESs to build their mathematical model. As a result, both the MLR and ANN models were able to predict viscosity with high accuracy (with an R^2^ value of 0.9305 for the MLR model and an R^2^ value of 0.9863 for the ANN model). Alrugaibah et al. [189] compared the usage of ANN and response surface methodology (RSM) models while investigating the efficiency of NADESs for extraction of procyanidins and anthocyanins from cranberry pomace. For extraction of anthocyanins using 8 NADESs under various conditions, the ANN models performed better than the RSM model (R^2^ = 0.95 for ANN vs. 0.88 for RSM). Fiyadh et al. compared two types of ANNs, namely feed-forward back-propagation (FFBP) and the layer recurrent (LR) networks for the prediction of lead (Pb^2+^) removal from water by DES-functionalized CNTs [190]. Through the utilization of the experimental data and implementation of the types of ANN models mentioned above, the authors established the influence of adsorbent dosage, the concentration of Pb^2+^, pH, and contact time (the input features) on the adsorption capacity of the DES-CNT adsorbent (the models’ output feature). After the optimization of the inner architecture of ANNs, the best prediction of lead removal was achieved by applying a feed forward back propagation (FFBP) ANN that gave a MSE of 1.66 × 10^−4^ and R^2^ = 0.9956. In another paper, Fiyadh et al. applied a NARX neural network (non-linear autoregressive network with exogenous inputs) for the prediction of arsenic removal from water using *N*,*N*-diethylethanolammonium chloride-based DES functionalized CNTs [191]. As in their previous work, they studied the effect of the same factors on the adsorption capacity of DES-CNTs. Using a NARX neural network with an optimized structure gave an MSE of 4.75 × 10^−4^ for the testing set (20 experimental data points) with R^2^ = 0.9922. In similar work, Fiyadh et al. explored the removal of the As^3+^ ions from water with benzyltriphenylphosphonium chloride based DES-CNTs by implementing a NARX-based approach [192]. The results showed that this model is suitable for the prediction of the adsorption of As^3+^ ions from water (R^2^ = 0.9818). Finally, in a separate work, Fiyadh et al. investigated removal of mercury ions from water using multi-walled CNTs functionalized with an allyl triphenylphosphonium bromide and glycerol-based DES [193]. After comparison of the NARX network, feedforward neural network and LR network models with optimized structures, it was discovered that the NARX model provides the best prediction of Hg^2+^ adsorption capacity with the R^2^ = 0.9701.

Dashti et al. used four ML models, namely particle optimization swarm (PSO-ANN), adaptive network-based fuzzy inference system (PSO-ANFIS), least-squares support-vector machine (LSSVM), and multi-variate polynomial regression (MPR). The models were trained and tested using a set of 333 experimental data to demonstrate their efficiency in the prediction of the CO_2_ solubility in different DESs [194]. It was shown that the LSSVM model can provide better performance and the highest accuracy with R^2^ = 0.993. In the work of Bagh et al., an ANN model was utilized for the prediction of electrical conductivity of ammonium and phosphonium-based DESs [195]. The ANN with 8 hidden neurons showed the best performance and gave the smallest R^2^ coefficient of 0.9988.

#### 2.6.3. Optimization of Experiments using ML

In addition to prediction of materials properties, ANN models can be used to design experiments, that is, they can identify optimal experimental conditions by analyzing the datasets containing information about how different factors (for example, temperature or humidity) influence the experimental outcome. As an example of such an application, Sharma and Dash utilized a combined ANN and genetic algorithm (ANN-GA) approach for how to establish parameters for a DES-based microwave-assisted extraction process (microwave power, extraction time, liquid–solid ratio, and water percentage in DES) that allowed achieving a high extraction efficiency of phytochemical compounds from black jamun pulp [196]. Stupar et al. applied the RSM and ANN model for the development of an optimized procedure for β-carotene ultrasound-assisted extraction from pumpkin using natural DESs [197]. Ghaedi et al. described the development of linear and quadratic regression models for the prediction of CO_2_ solubility in DESs and their aqueous solutions [198]. The authors used the designed quadratic regression model for investigating the influence of pressure, temperature, molar ratio, and water/DES concentration on the CO_2_ mole fraction and establishing the experimental conditions under which CO_2_ solubility in DESs and their aqueous mixtures reaches its maximum.

In the study by Xu et al., 42 key factors of DES pre-treatment of lignocellulosic biomass procedure were handled by principal component analysis (PCA) and partial least squares analysis methods to raise the possible efficiency of this industrial procedure [199]. Another case where PCA and regression analysis were used synergistically is the work of Kollau et al. [200]. In this study, the authors used a combination of experimental, theoretical, and computed properties as input for their linear and non-linear models to predict the non-ideality of the DES mixtures and, thus, the eutectic temperatures. As a result, the non-linear model with singular descriptors appeared to be significantly more accurate with R^2^ = 0.93.

#### 2.6.4. Different Aspects Regarding Application of ML Methods

One of the greatest benefits of the ML approach is that it can be combined with MD simulations. ML algorithms can be used not only for the construction of FFs, but also in post-processing of simulation data or/and their interpretation [201,202,203]. Moreover, the results of the MD simulation can be used as an input for ML models [184]. Although some works exist where MD and ML methods have been applied synergistically, to the best of our knowledge, there are currently no examples of the implementation of these two approaches in combination with DES research.

Despite the vast number of possibilities that ML methods offer, they have their own limitations. For example, during iterations, the algorithm can converge to a local minimum of the error function [182,194]. Moreover, in order to avoid the common problem of overfitting, some advanced ML models use the molar structure (e.g., graph-convolutional neural networks [204]) as their direct input. As a result, the number of parameters may be so large that they require a substantial amount of data to properly estimate the weights [184]. Thus, gathering a sufficient amount of experimental data is a major obstacle for developing advanced ML models, since training of these models may require thousands and even hundreds of thousands of entries on molecular properties that may not be available. A promising solution to this problem may be developing advanced ML models by applying the so-called “transfer learning” approach [205]. It implies a two-stage protocol of ML model learning: (1) pre-training using data on proxy-properties and (2) fine-tuning using data on the target property(ies). Passing the first stage of the protocol typically requires large “synthetic” (computationally obtained) databases (for example, QM9 [206], Open Quantum Materials Database [207], etc.). For this reason enlarging and developing “synthetic” databases specifically for DES is among the most vital tasks.

## 3. Main Directions of Investigations

### 3.1. DES Structure

One of the main aims is determining the DES structure-property relationship. Considering the huge number of conceivable combinations of possible DESs, insights into the properties of DESs at the nanoscopic level are critical.

**Figure 3:**
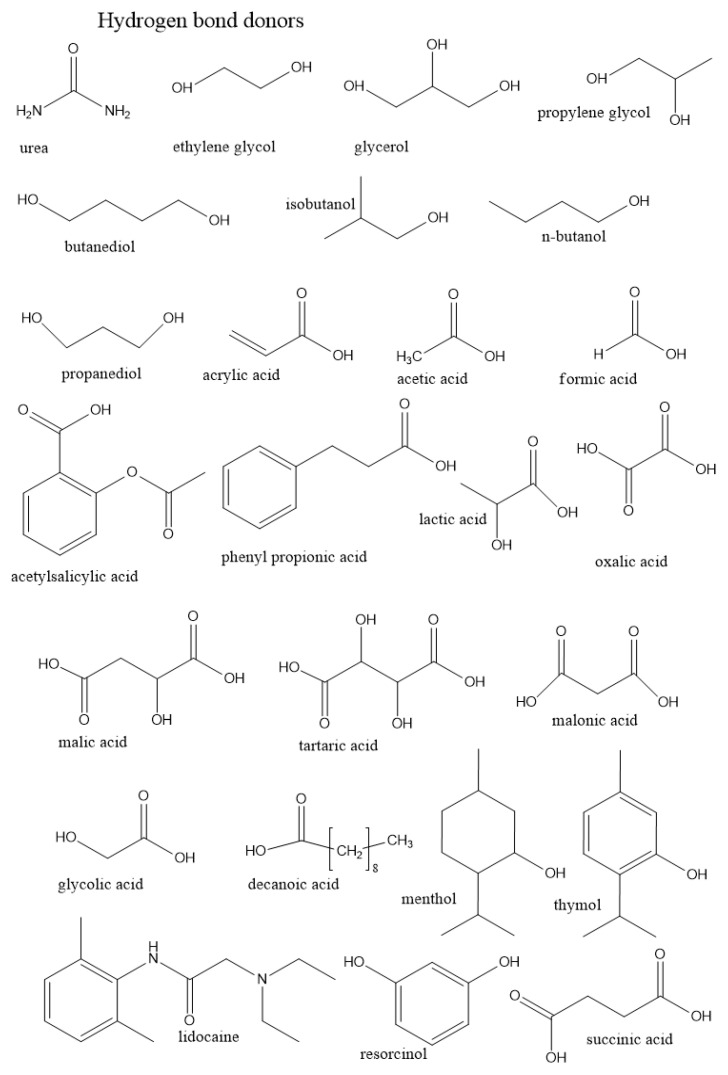
Chemical structures of H-bond donors of DESs discussed in this section.

**Figure 4:**
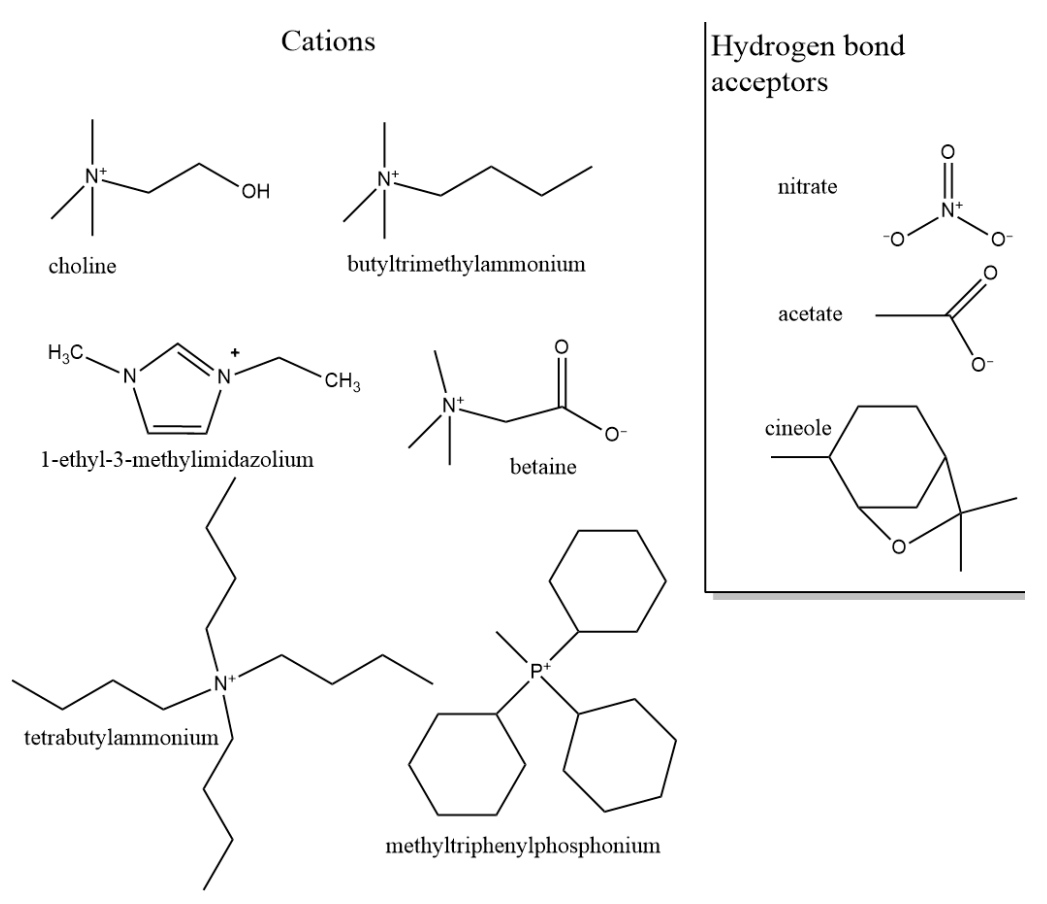
Chemical structures of cations and polyatomic H-bond acceptors discussed in this section.

In this section, we summarize the structural characteristics of different DESs obtained by different simulation methods. Most of the work has been devoted to the third type of DES (see Table 1 in Introduction). It consists of a HBD and an organic salt. To control the structure and properties of DES, each component can be varied. We first overview the role of each component in DES structure formation based on existing simulation studies. Figure 3 and Figure 4 illustrate the chemical structures discussed in this section.

#### 3.1.1. Role of the Hydrogen Bond Donor

Most of the works on the structures of classical DESs are based on ChCl, such as reline [64,70,76,123,208,209,210,211,212] (HBD is urea), ethaline [161,210,213,214] (HBD is ethylene glycol), glyceline [82,210,215] (HBD is glycerol), and propoline [216,217] (HBD is propylene glycol). Over the last five years, their structures and properties have been investigated intensively and the main interactions have been determined. In particular, the interplay of soft and strong interactions confers flexibility of the hydrogen-bond network formed in DESs and allows the ensemble to remain liquid at room temperature. Thus, for reline, urea molecules interact with Cl^−^ ions weakening their interactions with choline cations, which leads to a decrease in the melting point of the mixture [4]. The compounds for DESs should provide a competitive balance of interactions between them to ensure the depression of the melting point. The position of the eutectic point is dependent on the activity and the melting properties of individual DES components and their fraction in the mixture. The theory of solid–liquid phase behavior of a simple eutectic system was described by Alhadid et al. [218]

Celebi et al. [123] used MD simulations to accurately describe the influence of the fraction of urea in DESs based on ChCl and urea. They showed that the H-bond network between ions and urea molecules disappears as the mole fraction of urea increases. In addition, they demonstrated a non-monotonic behavior between the urea fraction and ionic conductivity: the latter increases with increasing urea concentration and reaches a plateau at reline composition. Shayestehpour et al. also recently highlighted the main molecular features responsible for the properties of ChCl/Urea mixtures using MD simulations [119]. In particular, they demonstrated the key role of urea in the formation of the H-bond network in reline.

Instead of urea, Bonomo et al. [215] investigated DESs based on ChCl and glycerol (which has three hydroxyl groups). They showed that in the case of glycerol 1:2 composition, the coordination is probably defective, and chloride stabilization is ensured both by H-bonding with the choline hydroxyl group and electrostatic interactions with the tetramethylammonium group. At 1:3 composition, the excess of glycerol was sufficient to stabilize chloride anions due to the high amount of hydroxyl group in them.

Stefanovic et al. compared three DESs based on ChCl, ethaline, glyceline, and reline by AIMD simulations [210]. They showed that the structure of the bulk HBDs is largely preserved for glyceline and ethaline which can explain a smaller melting point depression. In contrast, reline exhibits a well-established hydrogen-bond network between the salt and HBD, leading to a larger melting point depression. The extensive hydrogen-bond network in reline also results in higher viscosity compared to ethaline and glyceline. Glyceline also exhibits over-saturation of HBD groups, which leads to higher cohesive forces within the bulk liquid and to a higher viscosity than ethaline due to more extensive interactions between HBDs. Another comparison of the choline-based DESs was performed by Ferreira et al. [216,217]. In their first work [216], they developed a non-polarizable OPLS-based FF for propeline. The results showed that the HBDs in propeline have a preference to interact with the salt rather than with itself, which explains its relatively high viscosity. In the follow-up work Ferreira et al. [217] compared four DESs, namely, ethaline, propeline, propaneline (based on propanediol), and glyceline. Glyceline, which has a higher number of hydroxyl groups, demonstrated a higher degree of H-bonding formation with the anion. Despite the number of HBD groups, an important factor determining the DES properties is the size of the HBD molecule. Thus, the smaller molecular size of ethaline compared to propeline and propaneline, allows these molecules to become closer to choline leading to a higher density of DES based on diatomic alcohols. The critical role of the H-bond network in DES formation was also recently demonstrated using MD simulations by Panda et al. [219] who compared DESs based on tetrabutylammonium chloride and two different HBDs glycerol and ethylene glycol.

Role of HBD can be played by acids, which can endow a DES with unique properties [68,220,221,222,223,224]. Fu et al. [220] developed a DES based on acrylic acid and estimated the stability of the ChCl-acrylic acid complexes by QM calculations. The authors demonstrated a strong interaction between ChCl and acrylic acid, which is more stable than interactions between individual components. This is an important result because acrylic acid is able to polymerize, and a DES containing it has the potential to function as an ink for 3D printing. Gautam et al. [68] used DFT to compare the structure of clusters formed in DESs based on acetic acid and formic acids. The authors detected the formation of strong H-bonds between the hydroxyl groups of choline, chlorine ions, and double-bonded oxygens in carboxylic acids. It is important to note that the viscosity of DESs based on formic acid is two times lower due to its smaller size and faster movement in the liquid structure. The importance of the size of HBD was also shown by Rozas et al. [154] who used MD simulations and revealed the mechanism of H-bond network formation in salt-free cineole-based DES based on different acid HBDs: the interaction between cineole and HBD are highly dependent on the size of the HBD. Access to the ester group of cineole is sterically hindered, and only small molecules can form the most favorable interactions with it.

As discussed in Introduction, components of DESs could act as active pharmaceutical ingredients forming so-called therapeutic DESs. These DESs can be used to enhance the solubility of active ingredients, membrane transport, drug delivery, and bioavailability [225,226]. Saha et al. [222] used combined DFT/MD simulations to study the possibility of developing DESs based on acetylsalicylic acid (aspirin), and Bonab et al. [223,224] simulated DESs based on ChCl and phenyl propionic acid, which have a wide variety of uses including cosmetics, food additives, and pharmaceuticals [227]. The authors aimed to understand the physical mechanisms occurring at the eutectic composition point.

Polyols and acids have also received attention [67,228]. Naseem et al. [67] used MD simulations to compare DESs based on polyols (ethylene glycol and glycerol) and acids (malic acid, tartaric acid, and oxalic acid). The DES based on tartaric acid was found to be more stable due to the larger number of HBD groups in tartaric acid compared to other HBDs. The H-bond network, as revealed by QM simulations, showed a three-dimensional structure via cross-linking through carboxyl groups of tartaric acid and choline’s hydroxyl group. Similar results have been obtained by Perkins et al. [228] and Bruinhorst et al. [229]. Perkins et al. [228] showed that a DES based on malonic acid is much more stable than ethaline and glyceline. Bruinhorst et al. [229] simulated DESs based on heterocyclic amino acid proline as HBA and glycolic acid or malic acid as HBD and showed that malic acid with the largest number of HBD sites forms the most stable DES.

#### 3.1.2. Role of Hydrogen Bond Acceptor (Anion)

Another way to control the properties of a DES is to change the H-bond acceptor (HBA). Because HBA is involved in both interactions (with cation and HBD), its replacement will cause changes in both interactions. This makes property and structure prediction a non-trivial task. Migliorati et al. [230] recently discussed the role of an anion in H-bond network formation. They compared the structure and properties of DESs based on four different anions: chloride, fluoride, nitrate, and acetate. The results showed that there is no one-to-one correspondence between the order of DES melting points and the strength of the H-bonds between urea and anion; a complex network of interactions is formed in which the anions try to maximize their H-bond interactions with the other components of the system. The specific way in which each anion achieves this goal depends on the nature of the anion. It was shown that unlike monatomic anions, polyatomic anions, such as nitrate and acetate are able to simultaneously bind two hydrogens of urea.

#### 3.1.3. Role of Cation

Most of the studies regarding cations have focused on DESs based on ChCl. Migliorati et al. [211] compared structures of DESs based on choline (reline) and butyltrimethylammonium (UBTMAC). H-bonds between chloride ions and urea molecules are more favored in DESs based on UBTMAC due to absence of competition from the anion. This result suggests that the formation of anion-urea H-bonds is not the only reason for the large melting point depression observed in DESs, so a more complex picture has to be considered in which a variety of different H-bonds exists. In this context, it is worth mentioning the work of Gutiérrez et al. [153] in which a DES based on amino acid betaine and lactic acid was developed. Betaine has a close similarity in chemical structure to choline, where the hydroxyl group of choline is instead replaced by a carboxyl group. This produces a stronger H-bond network in DESs based on betaine and makes such DESs promising for future applications.

The role of another cation, 1-ethyl-3-methylimidazolium (EMIm), for (EMImCl]):urea DES structure formation was investigated by Cerajewski et al. [231]. Their MD simulations revealed nanoscale segregation of DES into two regions: EMIm and urea-enriched regions. The properties of the DES are determined by the interface between these regions, which depends on the interaction of chloride anions with urea and EMIm. Another example is the fact that one can control the structure of DES and its intramolecular interactions by varying cation types is the work of Naik et al. [232]. They demonstrated the difference in structure and formation of the H-bond network of DESs based on methyltriphenylphosphonium bromide (MTPPBr) and tetrabutylammonium bromide with ethylene glycol or glycerol as HBD [232]. It is worth mentioning that a DES based on MTPPBr is highly required for CO_2_ adsorption and its structure has been studied using MD simulations by Kussainova et al. [233].

#### 3.1.4. Hydrophobic Deep Eutectic Solvents

One of the actively developed types of DESs are hydrophobic DESs. Hydrophobic DESs were presented as solvents for liquid–liquid extractions in 2015 [234,235]. Since then, the field of hydrophobic DESs has grown extensively. The development of hydrophobic DESs is well described in the review by van Osch et al. [236].

Several authors have reported that the structure of hydrophobic DES could be heterogeneous. Because the structure of a DES is mostly determined by the H-bond network, the investigation of the influence and diversity of H-bonds on the structure is one of the common tasks. Thus, Salehi et al. [237] investigated the effect of the hydrophobicity of the cation on the structure and properties of the DESs. They performed MD simulations of DESs based on tetraalkylammonium chloride and decanoic acid with varying lengths of the alkyl side chain of the cation. The increase in cation chain length decreases the density and slows down diffusion. However, no significant influence was observed on the intermolecular characteristic distances and the H-bonds. Abbas et al. [238] investigated the structure and dynamics of H-bonds in hydrophobic salt-free DESs formed by the composition of decanoic acid, menthol, thymol, and lidocaine by MD simulations. They demonstrated the critical role of H-bonding on the structure and dynamics of DES and revealed a high diversity of H-bonds. The strength of the dominating H-bonds determines the diffusion of components in DES and the character of the H-bond network.

Control of heterogeneity is an important task for the development of DESs for extraction and other separation applications [238,239]. Alizadeh et al. [239] studied heterogeneity in DESs based on ChCl and its depravities with different lengths of the alkyl chain. MD simulations revealed strong heterogeneity caused by the segregation of polar and non-polar parts of molecules in DESs based on the cations with the long alkyl chains. A similar result was observed by Cui et al. [240], who compared the structures of DESs based on tetramethylammonium and tetraethylammonium, and Migliorati et al. [211] butyltrimethylammonium in the simulations discussed above.

#### 3.1.5. Electrolyte-Based DESs

Heterogeneity in structure has also been observed in electrolyte-based DESs (the first and fourth type of DES). Direct evidence of nanoscale spatial heterogeneity in electrolyte-based DES Li^+^/ClO_4_^−^:alkylamides (acetamide and propionamide) was reported by Kashyap et al. [241,242]. The authors demonstrated that nanoscale spatial heterogeneity is exhibited by the segregated domains of the constituent electrolyte; elongation of the tail of alkylamide enhances the extent of nanoscale morphology and the strength of ion-pairing. Moreover, they found that the degree of heterogeneity increases with temperature and explained this by the enhanced correlations between the ionic species that overpower the decrease in ionic species-alkylamide cross-correlations.

Biswas et al. [243,244] performed MD microstructure simulations for a number of Li salts (Li^+^, Br^−^, NO_3_^−^, ClO_4_^−^) and acetamide to identify the solution-phase microstructures in these media, and investigated the anion and temperature dependence of these microstructures. The authors showed that the presence of heterogeneity arises from the balance of the interactions between the various species. Ionic clusters were found to be most stable in the presence of NO_3_^−^. It was also revealed that the perchlorate DES is the most heterogeneous among the three systems studied.

New DESs based on lithium salts are promising electrolytes for lithium-ion batteries operated in low-temperature environments. In this context, the phenomenon of the decrease in melting temperature in metal salt-based DESs is relevant. Ogawa and Mori [245] combined MD and DFT methods to study four representative DESs based on LiCl or Lithium bis(trifluoromethanesulfonyl)imide (LiTFSI) and urea or tetramethylurea as HBDs. They compared directly the coordination states between Li salts and amides with or without NH groups, such as urea (with NH) or tetramethylurea (without NH), and revealed the eutectic mechanism of DESs. It was established that if the cation in the DES is bulky, such as in reline, the NH group coordinated with Cl^−^ ions causes the melting point to decrease. In contrast, in the case of high Lewis acidity of the cation (such as Li^+^), the CO group in amide coordinates preferentially with cation. In the case of DESs based on LiTFSI and an amide, the presence of an NH group may not lead to a decrease in the electrolyte melting point. Furthermore, the HOMO–LUMO calculated from DFT to estimate electrochemical stability showed that Li-salt:amide-based electrolytes with NH group are unstable on the reduction side. So, in contrast to ChCl-based DES, for lithium-ion batteries it is preferable to use compounds without any NH groups.

#### 3.1.6. Ternary DES

Recently, the possibility of the formation of ternary DESs (TDESs) has been put forth which offer lower viscosity and melting points than binary DESs.

The ternary DES choline chloride (ChCl):resorcinol (Res):glycerol (Gly) mixture was simulated using MD by Li et al. [246]. It was concluded that ChCl, resorcinol, and glycerol form numerous H-bonds that lead to the destruction of the intrinsic microstructure of each component. As a result, ChCl/Res/Gly are strongly associated through supramolecular H-bond network and form a DES.

The influence of alcohols as ternary components (n-butanol, iso-butanol, and butandiol) as an additional HBD in a binary DES composed of ChCl and malonic acid in equimolar ratio (1:1), also called as maline, was examined in Ref. [59] to understand the H-bonding interactions. The calculations focused on the molecular orbital (MO) energy levels. It was concluded that the H-bond network formed between maline and butandiol results in a larger melting point depression in comparison to n-butanol and iso-butanol. The interpretation was that maline and butandiol (in contrast to n-butanol and iso-butanol) form stable and homogeneous systems. These findings were further supported and significantly validated by the evaluation of the total energy. Maline:butandiol makes a homogeneous mixture to form a TDES with a less negative value, while n-butanol and iso-butanol with more negative value show prominent phase separation. The conclusion was also supported by the recent work of the same authors [247] where they calculated the MO energy levels for the molecular structures formed in TDES maline/butandiole. The results indicate that maline molecule more easily donates electrons accepted by the unoccupied orbitals of the two hydroxyl groups of the HBD.

Jangir et al. [60] studied the effects of alcohols such as ethanol and ethylene glycol as additional HBD (cosolvents) on DESs formed using ChCl as the HBA and l-lactic acid as the HBD at 1:2 molar ratio. The calculations of the MO energy levels revealed that the ethanol-based DESs showed more favorable hydrogen bonding than the ethylene glycol-based DESs leading to a thermodynamically stable binary system.

### 3.2. Dynamic Properties

Transport properties, namely diffusion coefficients and viscosity, are important parameters characterizing the potential of DESs practical applications. Significant attention has been dedicated to the investigation of mechanisms of motion of DES components, dynamical heterogeneities, as well as their dependence on different factors (such as ion identity, chain length, polarity, etc.). MD simulations could provide valuable insights into the dynamics of different components of DESs which is not easily possible in experiments. For example, quasi-elastic neutron scattering (QENS) experiments provide only ensemble-averaged results for the system [248]. Moreover, MD simulations allow access to smaller time and length scales, thus giving more information about dynamic properties and underlying mechanisms of motions in DESs.

Srinivasan et al. used MD simulations in addition to QENS to examine nanoscale dynamics in DESs comprised of acetamide (C_2_H_5_ON) with lithium nitrate (LiNO_3_) or lithium perchlorate (LiClO_4_) to investigate mechanisms of motion of their molecular components [248,249,250]. In particular, it was observed that movement of acetamide within the temperature range of 300 to 365 K consists of localized motions in transient cages formed by the neighboring molecules (both ions and acetamide) and cage-to-cage jumps. Thus, there are two types of acetamide molecules: (i) H-bonded to lithium ions (slow diffusion) and (ii) completely free of any H-bonds (fast diffusion). Interestingly, for the first type of acetamide molecules, jump diffusivity is at least 3 times lower and the mean residence time between jumps is twice larger than those for the molten acetamide. Additionally, it was found that almost all lithium ions (90%) are moving between the cages by a vehicular motion between solvation shells formed by 3–4 acetamide molecules. Only a small number of ions (10%) are diffusive due to the structural relaxation of cages. At the same time, no correlation was observed in the movement of ions, implying a system of dissociated anions and cations.

MD simulations were extensively used by Biswas’ group to prove the existence of dynamic heterogeneity in DESs [251,252,253,254,255,256]. Das et al. [251] applied MD simulations to provide support for the fractional viscosity dependence of rotation rates of fluorescence probes observed in experiments for DESs based on acetamide (C_2_H_5_ON) and lithium bromide (LiBr) with an acetamide mole fraction of 0.78. Analysis of the wavenumber-dependent incoherent and coherent scattering functions for acetamide molecules calculated at 303 K and corresponding to four different length scales of density fluctuations showed stretched exponential behavior. This provided evidence for temporal heterogeneity, thus explaining the experimentally observed fractional viscosity.

Guchhait et al. [252] simulated acetamide-based DESs with lithium perchlorate (LiClO_4_), lithium bromide (LiBr), and lithium nitrate (LiNO_3_) as electrolytes. Three systems with different mole fractions of acetamide (C_2_H_5_ON) and electrolytes were studied (0.81C_2_H_5_ON + 0.19LiClO_4_, 0.78C_2_H_5_ON + 0.22LiNO_3_, and 0.78C_2_H_5_ON + 0.22LiBr). The observed stretched exponential relaxation of the dynamic structure factors (even at ~150 K above T_g_) allowed explanation of viscosity decoupling in terms of the temporal heterogeneity of the DES medium controlled by anion identity.

A more comprehensive molecular view regarding the influence of anion identity on orientational jumps was given by Das et al. [253] who carried out MD simulations of DESs composed of acetamide and different lithium salts (bromide (Br^−^), nitrate (NO_3_^−^), and perchlorate (ClO_4_^−^)) with 78:22 mol ratio of the components at 303 K. Orientational jumps involve a bifurcation of a H-bond, switching of a binding partner followed by a large-angle rotation of the molecule. This mechanism was first suggested (using MD simulations) by Laage and Hynes [257] for water and has since been shown to be an important mechanism in solvation especially in the presence of hydrophobes [258]. To study the orientational jumps of DES components, Das et al. analyzed both acetamide-acetamide and acetamide-ion pairs. Their analysis of the MD data showed that: (1) Compared to the other two anions, the presence of NO_3_^−^ leads to less frequent large-angle jumps; (2) compared to NO_3_^−^ and ClO_4_^−^, the presence of Br^−^ anion has a different impact on the jump angle distribution, resulting in a bimodal form; (3) the energy barriers of orientational jumps of acetamides H-bonded to NO_3_^−^ and to ClO_4_^−^ differ almost by a factor of two; (4) viscosity of DESs has an opposite trend compared to the relative reorientational jumps displacements (both radial and angular) of sequence ClO_4_^−^ > NO_3_^−^ > Br^−^; and (5) there is almost no difference between the free energy barriers of orientational jumps for acetamide-acetamide in systems with different anions, the value being also close to that for molten acetamide. Additionally, the presence of dynamic heterogeneity in the systems was supported by the fact that jump time distributions exhibit a power-law dependence for all the anions studied.

Interestingly, the opposite conclusions about dynamic heterogeneity of DESs were made by Das et al. [254] while studying systems composed of acetamide (C_2_H_5_ON) + urea (CH_4_ON_2_) at 338 K with acetamide mole fractions of 0.6 and 0.7. These systems are non-ionic DESs composed of dipolar molecules and amphiphiles. Examining the mean squared displacement profiles, heterogeneity parameters, displacement distributions, and relaxation of dynamic structure factors showed that acetamide + urea is both a spatially and dynamically homogeneous system.

Mukherjee et al. [255] examined systems based on acetamide + urea. Pure systems containing only urea molecules were also simulated for reference. By analyzing various correlation functions, the authors proposed an explanation for the physical origin of the three slowest time scales measured in dielectric relaxation. They suggested that they are dominated by structural H-bond relaxation that involves center-of-mass translation. Particularly, they proposed that the origin of the fastest time scale in dielectric relaxation that lies in the sub-10 ps regime could be fast reorientational dynamics of the components (acetamide and urea). Additional conclusions were made about the time and length scales of dynamical and spatial heterogeneity in DESs, which were estimated to be on the order of ~10 ps and ~10 Å, respectively.

Reorientation dynamics was also studied by Rajbangshi et al. [256] using MD in ChCl + urea DES. They simulated systems at 0.33 mol fraction of urea at six different temperatures between 293 and 333 K. Their results suggested evidence for strong temporal heterogeneity in DESs based on, e.g., displacement distributions and dynamic susceptibilities. The comparison of a rank-dependent average reorientation relaxation time and translational diffusion also provided support for translation-rotation decoupling.

The above works use a single FF in MD simulations, the most popular ones being OPLS-DES [116] (in Ref. [256]), CHARMM [259,260,261] (in Refs. [248,249,250,251]) and its modified versions [261,262,263] (in Refs. [248,249,250,252,253]) and combined versions (CHARMM [259,260,261] + GROMOS [264]) (in Refs. [254,255]). The atomistic models in these works were mostly validated by measuring density [252,253,254,255,256] and good agreement was found. The situation is different for dynamic properties. Despite partial agreement with experiments regarding viscosity [249,255] and ionic conductivity [249] at a single state point, the temperature transferability of dynamical properties suffers from a lack of quantitative agreement. For example, Rajbangshi et al. [256] have shown that the ratio of diffusion coefficients measured with pulsed-field gradient nuclear magnetic resonance to those estimated in MD simulations lay in the range of 0.58 to 4.82 for choline and 0.63 to 4.76 for urea in the temperature range 293 to 333 K, despite using a specific OPLS-DES FF [116]. This result emphasizes the importance of FF development and inclusion of polarizability.

Perkins et al. [228] examined the influence of different FF parameters on various properties of the most commonly investigated DESs based on ChCl and urea using a single molar ratio 1:2. The authors showed that using the default values of GAFF and charges reduced by a factor of 0.8 (HF/6–31 G* level calculation) provides a better agreement with experimental data for density, thermal expansion coefficients, and heat capacity compared to other FFs. They found the self-diffusivities of the DES components at 298 K are underestimated between 25 and 51% compared to experimental data. On the other hand, good agreement with experimental values was observed at 330 K.

In their follow-up work, Perkins et al. [124] found that better agreement with experimental values of diffusion coefficients could be achieved by reducing the partial charges by a factor of 0.9 in the ionic species. This result was shown for three ChCl-based DESs: ChCl + ethylene glycol 1:2 (ethaline), ChCl + glycerol 1:2 (glyceline). They found that at 298 K the diffusion coefficients were underestimated only by 20–30% (for ethaline) and by 14–20% (for glyceline). At 330 K the discrepancy was 5–25% (for ethaline) and 17–27% (for glyceline).

The above shows the complexity of finding universal charge scaling and parameterization for different DES systems. For a more comprehensive discussion on simulations of DES transport properties, we refer the reader to the excellent review by the group of Smirnova [41].

To conclude, an alternative way to achieve more accurate predictions could be via the use of polarizable FFs. A recent example is given by Goloviznina et al. [138], who used the polarizable CL&Pol FF to simulate transport properties of ChCl + ethylene glycol (1:2 molar ratio). Their results show good agreement with experimental data at 298 K (2.18 × 10^−11^ m^2^ s^−1^ in MD vs. 2.62 × 10^−11^ m^2^ s^−1^ in the experiment) and viscosity (35 ± 5 mPa·s in MD vs. 37 mPa·s in the experiment). However, the H-bond donor diffusion coefficient was significantly overestimated (13 × 10^−11^ m^2^ s^−1^ in MD vs. 4.77 × 10^−11^ m^2^ s^−1^ in the experiment). Improved performance of CL&Pol compared to its non-polarizable version (CL&P) was demonstrated for the diffusion coefficient of ethylene glycol in DESs based on ChCl (1:2), as shown by de Souza et al. [140]. Interestingly, a better agreement between MD and experimental results was achieved when using temperature-grouped Nosé–Hoover thermostats rather than traditional ones [138]. According to the authors, the reason for that is a better treatment of translational, intramolecular, and polarization degrees of freedom.

### 3.3. DES for Separations and Gas Capture

One of the world’s major challenges is the reduction in greenhouse gases. Acid gas emissions, especially CO_2_, are one of the most pressing technical challenges of this century, given their role in driving climate change and ocean acidification. The world’s CO_2_ emissions are emitted in a number of ways, such as burning of oil, coal, natural gas, or liquid gas in power plants, or for instance by aluminum or petrochemical industries. Despite recent advances and developments in renewable energy sources, it is likely that at least for the next few years, fossil fuels will continue to play a key role in energy production. This means that CO_2_ emissions will inevitably continue to increase [265,266,267]. Thus, developing more sustainable and environmentally friendly ways to capture CO_2_ (before, during, or after processing) from fossil fuels is a major challenge today.

A wide range of CO_2_ capture technologies have been proposed over the past few years, including solid and liquid sorbents and sorption through membranes [268]. Selective membrane separation technology is one of the most promising methods and it is considered to be a cost-effective method to mitigate the emission of CO_2_. It is necessary to use materials that can effectively separate and capture gases on industrial scales. Membranes based on DESs are highly promising due to their unique properties and relative cheapness [269].

Another challenge is the removal of carbon dioxide from natural gas. Natural gas exists in deep underground reservoirs as a shale gas with non-hydrocarbon components, such as CO_2_. The presence of CO_2_ in natural gas is undesirable due to corrosion and low heating value; one of the difficulties in using natural gas is the removal of CO_2_ from it [270,271].

García et al. [268] used MD simulations to study the intermolecular interactions of different DESs (reline, glycine and maline) in contact with gas phases consisting of pure CO_2_, pure SO_2_, and a model flue gas (containing N_2_, CO_2_, O_2_, and water). It has been established that their intermolecular interactions depend on the nature of H-bonding sites available in the HBDs. Mechanisms of CO_2_ absorption were also investigated on methyltriphenylphosphonium (MEA) bromide and mono ethanol amine-based DESs using MD simulations by Kussainova et al. [272]. The authors found that interactions between the CO_2_ molecules decreased significantly in the presence of the DESs, while interactions between CO_2_ and MEA became enhanced. In addition, strong interactions between Br^−^/CO_2_ and MEA/CO_2_ were shown, which suggests the predominant sorption of CO_2_ by these components. Haider et al. [273] investigated the CO_2_ removal process from model shale gas using two DESs, reline and ethaline. They found that the process of CO_2_ recovery from shale gas with a DES is better than the conventional methyl diethanolamine (MDEA)-based acid gas removal process.

Investigation of highly CO_2_-philic DES-based separation membrane was carried out by Lin et al. [274]. The membrane was developed by nano-confining ChCl/ethylene glycol (ChCl/EG) DES into graphene oxide nano-slits. Their MD simulations revealed that the confinement affects the structure of the nanosized ChCl/EG liquid, which greatly facilitates CO_2_ transfer. It has been shown that by adjusting the ChCl/EG molar ratio and membrane thickness, it is possible to create materials with desired properties, which makes it a promising membrane for the selective separation of CO_2_. Similar studies were carried out by Shen et al. [275]. This study was aimed to understand how slit-like nanopores of graphite and titania (rutile) walls containing different amounts of DESs would perform in the gas binary mixture of CO_2_ and CH_4_ separation. Such a system is relevant for the separation of carbon dioxide from methane in natural gas. Lin et al. proposed a new kind of supported liquid membrane by incorporating a DES (1ChCl:4EG eutectic liquid) into the nano-slits of titanium carbide (Ti_3_C_2_T_x_) membrane [276]. Their MD simulations were applied to the resulting Ti_3_C_2_T_x_-based deep eutectic liquid membrane which showed a good preference for CO_2_ in permeability, selectivity (over other light gases), high heat resistance, and durability.

Alioui et al. [277] used MD simulations and a theoretical approach to study the molecular interaction between CO_2_ and different DESs. A relationship between the solubility of gas molecules and the energy of their interaction with DESs was established: The solubility of CO_2_ in DESs becomes greater when the energy of attraction is higher and vice versa [277]. Wang et al. [278] studied phosphonium-based DESs and found good agreement with experimentally determined solubility coefficients.

In addition to the problems described above associated with capturing carbon dioxide from the processing of fossil fuels or removing CO_2_ from natural gas combustion, desulfurization is a critical process for producing quality fuel. This required researchers to develop new and environmentally friendly methods for fuel desulfurization. Research shows that DESs have provided a new route for fuel desulfurization due to the cheapness and availability of raw materials, higher desulfurization efficiency and environmentally friendly properties [279]. Hydrodesulfurization and Extractive Desulfurization are among the most promising desulfurization methods due to their simple operation, low-cost, and high efficiency when using low-quality fuels [279,280].

MD simulations could be used for the theoretical investigation of these desulfurization processes by DESs: in the study by El-hoshoudy et al. [280], it was shown that DESs can be used to capture and remove thiophene compounds. Li et al. [279], proposed metal ion-based DESs (MDESs), which have even higher sulfur extraction efficiency, and Shah et al. [281] used MD simulations to investigate them. The authors showed that an MDES based on tetrabutyl ammonium chloride (TBAC), polyethylene glycol (PEG), and ferric chloride (FeCl_3_) could be useful in desulfurization of diesel and capable of rapidly removing thiophenic compounds, such as benzothiophenes and thiophenes. It was also shown that PEG-free systems can have higher extraction abilities than TBAC + PEG + FeCl_3_ systems.

In addition to the absorption of CO_2_ and SO_2_, and natural gas purification, DESs could also be used for extraction of pure components (aromatic and aliphatic) from naphtha streams. This is of great significance for the petrochemical industry due to the high economic value of its components [282]. Kumar et al. [282] investigated the molecular mechanisms of benzene extraction from hydrocarbon mixture using a phosphonium-based DES using MD simulations. They found that the van der Waals interactions prevailed over electrostatic ones and enabled the extraction of benzene from a DES-benzene-hexane mixture. In this ternary system, the DES-benzene pair had a higher interaction energy than DES-hexane. The self-diffusion value suggested a higher miscibility with DESs and benzene compared to hexane.

It should be noted that fossil fuels also contain a variety of nitrogen-based polyaromatic compounds in various forms. The presence of nitrogen-based polyaromatic hydrocarbons (PAHs) in fuels, which emit NO_x_ into the environment during fuel combustion in engines and industrial plants, have a large negative impact on the environment and ecosystem. Recent studies have shown that DESs based on phosphonium have a very high ability to remove PAHs from fuel oils. Naik et al. [283] studied the extraction of polyaromatic hydrocarbons from fuel oils using the DES-quinoline-heptane ternary system. The results of their MD simulations, similar to results obtained by Kumar et al. [282] showed that the van der Waals interactions were the controlling interactions. Thus, the low-cost DES could be used for extraction of PAH from fuel oils.

Oxygen-containing additives are widely used in the production of gasoline to reduce lead content and thereby minimize the severe engine knocking induced by hydrocarbon mixtures in gasoline. For the production of these oxygenated additives, more and more processes are being developed for the coexistence of alkanes resulting in many azeotropic mixtures (such as *n*-hexane-ethanol and *n*-heptane-1-butanol). However, these azeotropic mixtures are difficult to separate by conventional distillation. Liquid–liquid extraction (LLE) can be applied to solve this problem. In this case, DESs can be used as an extractant. The study by Zhang et al. [284] explored a choline-based DES for extracting 1-butanol (a renewable high-energy biofuel) from an alkanol azeotrope system. Simulations of the LLE process were performed using the MD method to explain the azeotropic separation extraction mechanism at the molecular level. The results showed that a ChCl + urea DES had the best extraction effect. Moreover, the results showed that the extraction of different DESs depends on the HBDs in DESs, and urea had the best performance among the studied HBDs. It was also found that among the three components of DESs, Cl^−^ ions played a dominant role in the extraction process. In addition to studies of 1-butanol + DES mixtures, there are investigations of other alcohol + DES mixtures separation. Verma et al. [19] first used non-ionic DESs to carry out extraction of ethanol, 1-propanol, and 1-butanol from the aqueous phase. MD simulations helped them to establish that the energy of interaction between DES and alcohol is much higher than between DES components and water, or water and alcohol. This fact explained the effectiveness of DES for alcohol extraction. Liu et al. [285] used MD simulations to investigate molecular mechanisms of separation of methanol: n-hexane mixture by ChCl based DESs and determined the most effective DES for the separation, and also showed the key role of Cl^-^ ions in the separation process.

Thus, today there are significant hurdles to face in the purification of various energy sources (fuel, gas) from different substances, such as acid gases, organic compounds, and others. Their presence can have a disastrous effect on the environment, polluting the atmosphere and on the quality of the fuel itself, from which these impurities were extracted. DESs can be used as separating membranes in the processes of gas (liquid) purification. In addition to pure DESs, some nano-confining (nanoparticles, such as graphene oxide or the nano-slits of titanium carbide) or additives of the MoO_3_ type can also be used for changing the properties of the gas separation membrane. Each method allows one to improve certain characteristics that are necessary for a specific industrial task. However, the processes occurring at the nanoscale are often completely inaccessible by experimental methods. In this regard, computer simulations are an excellent tool that allows studies of the molecular mechanisms.

### 3.4. Water Effect on DES

One of the important questions is the interactions of water with DESs. The effect of water on DESs and ILs has been reviewed by Ma et al. [35]. In practice, the presence of trace amounts of water in DESs is unavoidable in most cases [34,35,36]. However, even trace amounts of water can affect the H-bond network and significantly change the properties of a DES [35,36]. Water molecules have the ability to be both HBDs and HBAs and can therefore significantly modify the arrangement of DES at the molecular level [34,286].

Water can also be used to change many of the crucial properties of DES, such as viscosity, density, and ionic conductivity, to lower the financial cost and to preserve the environmental friendliness of the solvent [35,286]. For example, high viscosity is considered as one of the drawbacks of DESs that may impede their wider application [35,287,288] and increasing water content leads to a decrease in DES solution viscosity that is often a desirable effect [289,290].

#### 3.4.1. Water Effect on DES Micro- and Nanostructure

Much effort has been put into understanding the influence of water on the micro- and nanostructures of DESs [34,66,109,287,291,292,293,294,295,296,297,298]. Using MD simulations along with NMR spectroscopy, Di Pietro et al. investigated ChCl:urea and ChCl:glycolic acid DESs upon aqueous dilution [34]. The addition of water caused the displacement of DES components and asymmetric hydration around the Cl^−^ ions until water became the main ligand. Busato et al. analyzed the effect of water on the structure of ChCl/sesamol 1:3 DES [296]. It was shown that for water/DES molar ratios greater than 6, water segregates from sesamol and captures the majority of ChCl in the aqueous area. Weng et al. described the dual effect of water on DESs made of 1:2 ChCl/glycerol using MD simulations [287]. With the addition of water, the number of ChCl–glycerol supramolecular complexes in DES and the number of H-bonds between choline and glycerol decreased significantly. Water can also link choline to glycerol instead of chloride. Alizadeh et al. performed AIMD simulations to study ChCl:ethylene glycol DES structure with the addition of water (1:2:1 ChCl:EG:water) [294]. The results showed that water molecules compete for association with Cl^−^ anions. At the same time, some charge transfer occurs from the anion and the hydroxyl group of the cation to water.

QM calculations can also be used to complement experimental data for water–DES systems [66,293,297]. For instance, Faraji et al. compared molecular interactions in aqueous solutions of NADESs based on different amino acids [66]. It was shown that NADES containing lactic acid/histidine have the highest interaction energy compared with those NADESs based on alanine and glycine.

#### 3.4.2. Effects of Water on Reline

A large number of studies have focused on the structure and properties of reline in the presence of water [109,286,292,295,298,299]. Kumari et al. demonstrated that increasing hydration levels leads to the reduction in interactions between the components of reline while water preferentially solvates Cl^−^ anions, as well as hydroxyl and ammonium groups of choline cations [286]. Above 41 wt% of water content, the structure of reline changes dramatically and a transition from reline to an aqueous solution of reline components occurs. Below this point, the DES structure is qualitatively retained. More recently, Sapir et al. analyzed the effect of water on the nanostructure of reline using MD simulations [292]. The results showed that the nanostructure changed even at very low water content. Moreover, hydrated DES can be deconvoluted into two dominant nanostructures that prevail up to 30 wt% water: water-in-DES with preserved structural characteristics of pure DES, and DES-in-water where aqueous solvation of chloride and formation of water–chloride aggregates occurs. Thus, DESs in the presence of water are mostly heterogeneous, i.e., composed of a few structures. However, when water content is >50 wt%, a dilute aqueous solution of DES with solvation of the choline–chloride ion pairs is observed. The quantitative difference between water content that corresponds to a transition to a dilute aqueous solution obtained by Kumari et al. [286] and Sapir et al. [292] could be explained by the differences in the water models used in these works. Sarkar et al. [300,301] confirmed the results obtained by Kumari et al. [286] and Sapir et al. [292] and investigated changes in water structure with the increase in the fraction of reline [300] and pure ChCl [301]. They showed that in the presence of reline, water–water contacts are replaced with contacts between water and urea molecules and chloride anions, which affects water structure significantly.

Fetisov et al. [109] also demonstrated the micro-heterogeneous structure of reline DES and water mixtures using AIMD simulations. Similarly, results indicated that water preferentially solvates Cl^−^ anions. Furthermore, it was shown that the hydrogen atoms of urea have a similar tendency to bond to the Cl^−^ anions as well as to the oxygen of urea and water. Using MD simulations, Celebi et al. investigated the microscopic structure and thermophysical properties of aqueous reline and ethaline DESs [298]. It was shown that higher water content corresponds to more H-bond networks in reline and ethaline disappearing. Consequently, DESs were fully dissolved at 40 wt% of water. This corresponds to the results obtained for reline by Kumari et al. using the same SPC/E water model [286]. Alterations in DESs structures strongly influence their properties. With increasing water concentration, viscosity, and density of reline and ethaline decreased, self-diffusion coefficients increased while the ionic conductivity increased up to 60 wt% of water followed by a decrease.

Recently, Celebi et al. analyzed the thermal conductivity of aqueous solutions of reline, ethaline, and glyceline using non-equilibrium MD simulations [299]. Almost a doubling of the thermal conductivities was observed for all of the aforementioned DESs in the case of the addition of 25 wt% of water. The increase in water fraction up to 75 wt% leads to a three-fold increase in thermal conductivity. Bezerra et al. explored the effect of water on the electrochemical behavior of Ag^+^ ions in reline by combining experiments and MD simulations [302]. Using the cyclic voltametric technique, it was demonstrated that the addition of water catalyzes the electrochemical reduction in Ag^+^ ions in reline DES. MD simulations revealed structural features in the investigated mixtures upon addition of water, the number of urea molecules around the Ag^+^ ions slightly reduced while the water molecules adjusted to the free space in the DES. Thus, the results obtained in different studies for reline-based systems in presence of water are qualitatively consistent. The quantitative differences could be related to the differences in the particular water models used in these studies.

#### 3.4.3. Water’s Effect on the Properties of DESs

Computer simulations are widely used to analyze the effects of water on DES, which is important for practical applications [298,299,302,303,304]. Bezerra et al. [302] studied the effect of water content on the electrochemical properties of the Cu^2+^ ions in an ethaline DES. An increase in water content in ethaline led to an increase in the diffusion coefficient of the Cu^2+^ ions. Moreover, the addition of water electro-catalysed the electrodeposition of Cu on the Pt electrode. MD simulations allowed supplementing the experimental findings to understand the relationship between these properties and the structure. For instance, Bezerra et al. [302] demonstrated the complexation of Cu^2+^ ions with water molecules in DES. It was also shown that when the amount of water was less than 1%, Cu^2+^ diffusion remained almost unaltered.

Baz et al. [304] studied thermophysical properties of glyceline DES in aqueous solutions using MD simulations. The results showed that an increase in water content reduced the viscosity of the DES, while the thermodynamic activity of water increased. It was suggested that MD simulations can be used to predict the optimal composition with low viscosity and low enough water activity to be of importance for biocatalytic applications. Lukaczynska-Anderson et al. [303] studied the complexation of Ni^2+^ in 1:2 ChCl:urea (reline) and 1:2 ChCl:ethylene glycol (ethaline) and demonstrated that the addition of water changes the complexation of metal cations (Ni^2+^) which is reflected in electrochemical performance of DESs. Moreover, the addition of 0–10 wt% of water to reline led to a strong decrease in viscosity and an increase in conductivity. Interestingly, ethaline appears to be less sensitive to water addition than reline.

#### 3.4.4. Activity and Stability of Enzymes in DES/Water Mixtures

DESs can be also used as a non-toxic and biodegradable reaction medium for redox biocatalysis [288]. The stability and activity of enzymes in DES with different water concentrations have been addressed using MD simulations [288,291,305]. Kumari et al. [291] showed that the conformation of hen egg-white lysozyme is substantially destabilized in reline/water mixtures especially at 50:50 reline:water content. Huang et al. analyzed the activity and stability of alcohol dehydrogenase in glyceline/water mixtures [288]. At 10% of water content, the molecular flexibility of the enzyme increased which, in turn, can influence the enzymatic activity. At the same time, Shehata et al. showed that slightly hydrated reline (5%) activates thermoalkalophilic lipases while the mobility of the lid domain that controls catalytic activity increases [305].

#### 3.4.5. Hydrophobic DESs

Until recently, synthesized DESs were mostly hydrophilic and showed substantial solubility in water [306]. Increased hydrophobicity of DESs extends the range of their applications. For example, hydrophobic DESs can be used for separating toxic or important products from water.

There is an increasing interest in the synthesis, analysis, and application of hydrophobic DESs, which were first synthesized by van Osch et al. in 2015 [234]. Recently, Paul et al. studied the water stability of various hydrophobic DESs, tetrabutylammonium chloride-based DESs and menthol-based DESs with different organic acid-based HBDs [219]. MD simulations demonstrated the key role of H-bond strength on water stability. H-bond strength was related to the DES structure and the length of the alkyl chain of the HBDs. It was shown that DESs based on menthol and higher fatty acid (C8–C12) are water-stable. For menthol-based DESs, the order of stability was the following: dodecanoic acid > decanoic acid > octanoic acid > hexanoic acid > pyruvic acid > butanoic acid > levulinic acid > acetic acid, for ammonium-based DESs the order corresponded to the same sequence. The same order was demonstrated for the average number of H-bonds between HBDs and HBAs in DESs. Using MD simulations, Paul et al. investigated phase separation in a DES made of a 1:1 mixture of oleic acid and lidocaine in an aqueous solution [306]. It was found that the H-bonds and non-bonding interactions, as well as the competition between them play a crucial role in the phase separation process. Phase separation at higher temperatures was defined by the increase in unfavorable interactions between the DES and water molecules. In their recent work, Salehi et al. [307] studied the interfacial properties of the following hydrophobic DESs with water at different temperatures: tetrabutylammonium chloride–decanoic acid (TBAC-dec) 1:2, thymol-decanoic acid (Thy-dec) 1:2, and dl-menthol-decanoic acid (Men-dec) 2:1. Using MD simulations, they found that the hydrophobicities of the DESs did not depend significantly on temperature. The preferential alignment of oxygen atoms of decanoic acid toward the water phase was also indicated by large peaks on the density profiles. Thy-dec and Men-dec demonstrated strong hydrophobic behaviors with no leaching of the DES constituents into water and negligible water-in-DES solubilities.

#### 3.4.6. Prediction of DES/Water Mixtures Properties by ANNs

Special attention should also be paid to the application of ANNs for the prediction of DES/water mixtures properties. For instance, Fiyadh et al. developed ANNs to predict the removal of Pb^2+^ from water by DES-functionalized carbon nanotubes [190]. The following experimental variables were used as input parameters: adsorbent dosage, initial concentration of metal ions, pH, and contact time while the single output parameter was adsorption capacity. The ANN model was successfully used for prediction and the optimal topology of the neural network was found. Fiyadh et al. also studied the adsorption capacity of DES-functionalized carbon nanotubes for arsenic removal from water solution using ANNs [191].

### 3.5. DES in Nanotechnology

Understanding how the molecular-level structure relates to the properties of the solvent is critical to the design and development of DESs for commercial and industrial use. Over recent years, there has been a dramatic increase in computer simulation studies of interactions of DES components with nanoparticles of different chemical structures, molecular composition, mass fraction, and other properties using detailed fully atomistic computer simulations [61,65,87,96,308,309,310,311,312,313,314,315,316,317,318,319,320,321,322,323]. Generally, these studies have been carried out using atomistic MD methods [308,309,310,311,312,313,315,317,318,319,320,321,322,323,324] and quantum chemistry (QC) [61,65,87,96,314,316].

To date, DESs have attracted attention for their ability to break agglomeration of carbon nanoparticles, influencing their modification processes, and creating complexes between DES components and nanoparticles. Using atomistic computer simulations, the structure of DESs have been investigated close to the surfaces of different nano-objects: single-walled carbon nanotube (SWCNT) [324], graphene [308,310,313,323], nano-surfaces [308,310,313,315,317,320,322,323,324], nanopores [309,311], and metal nanoparticles [312,315,317,318,319,320].

As discussed earlier, one of the most common DESs is reline. The orientation of components of reline near the nanoparticle surface has been investigated using MD [310,313,316,322,323,324] and QC [316] modeling. The results show that in DES solutions, both HBA and HBD molecules are oriented in different ways. Several studies have also shown [313,317,318,324] the emergence order of DES components close to the interface. Pair distribution functions and densities between the nanofiller atoms and DES components show several peaks, characterizing the appearance of regions with different packing densities of DES components near the filler surface. In the vicinity of the nanoparticle surface, one can distinguish the appearance of one well-defined near-surface layer of DES components, which has a structure different from the one observed in the DES liquid state without contact with nanoparticle surfaces.

Lawal et al. [96] combined theoretical calculations and experimental measurements of adsorption of phenol and crystalline violet dye on carbon nanotubes modified by a DES (methyltriphenylphosphonium bromide and glycerol). At the molecular level, the interactions occurring between the surface of the SWCNT with phenol or crystalline violet dye in contact with the components of the DES were studied using QC methods. The authors concluded that the suggested DES could be used for nanofiller modification [96].

Wu et al. [61] studied the effect of bromine and Cl^−^ anions on how solvation occurs and supercapacitors’ characteristics. They showed that DES electrolytes based on tetraethylammonium bromide (TEAB) or tetraethylammonium chloride (TEAC) as the HBA and ethylene glycol (EG) as the HBD modulate how ion transport depends on temperature and electrode surface desolvation from activated carbon. In particular, their QC calculations showed that after DES formation with EG, the TEAB and TEAC LUMO energies become slightly reduced. Alternatively, HOMO energies decline more crucially in DES systems, which decreases electron loss and oxidation. The large differences between the LUMO-HOMO energies lead to the deterioration of electronic transitions and enhancement of complex stabilization.

Patidar et al. [316] characterized the amphiphilic star block ethylene oxide propylene oxide block (T1304 star) copolymers in different DES–water mixtures in solution by varying the molar water ratio. The results led to the conclusion that among the DES pool studied, glycerol with ChCl in DESs had the most prominent interactions with the T1304 star copolymer. The authors explained it by the fact that the large energy difference between HOMO and LUMO for a DES leads to the most stable solvent at room temperature. Using DFT, Ghenaatian and coworkers [87] studied clusters of metal particles (Cu, Ag, and Au) with DES comprising ChCl and urea. Analysis of the binding energies between the metal particles and DES shows that ChCl:urea interacts more with Au and less with Cu and Ag nanoparticles. With help of DFT calculations, Shakourian-Fard et al. [314] studied different ChCl-based DESs with graphene molecules (GNF) and graphene with defective double-vacancy and Stone–Wales forms (DV-GNF and SW-GNF). The results show that graphene defects lead to DES adsorption enhancements in the following order: DV-GNF > SW-GNF > GNF. The authors also found that the presence of aromatic fragments in DES enhances the van der Waals interactions with surfaces.

MD simulations are being extensively used to predict structural properties of DESs in the vicinity of nanoparticles. Shen et al. [309] modeled four DES systems of mixtures of choline iodide and glycerol at a molar ratio of 1:3, confined inside slotted nanopores whose walls are made of TiO_2_ or graphite. The limiting effect of the pore was found to be strongly influenced by the dominant arrangement of glycerol over DES cations and anions in the first near-surface layer close to the pore. The limiting effect of the wall considerably slows down the mobility of the DES components near the slit walls. Atilhan et al. [319] studied the solvation of various metal nanoparticles (gold, silver, etc.) in various DES solutions by all-atom MD methods. They observed the formation of two solvation layers surrounding the nanoparticles for all types of metals and DESs. In the first layer, intermolecular bulk interactions between the HBDs and metal atoms dominate, and interactions between anions and cations are almost absent. In the second layer, the concentration of their components is close to that of the DES solution. The study performed by Atilhan et al. [319] shows the promising use of different DESs for the solvation of various metal nanoparticles. They showed that due to their ability to stabilize nanoparticles and prevent their aggregation, DESs can be used as prospective solutions for the development of new nanoparticles with controlled properties.

Rozas et al. [313,324] investigated the influence of differences in the chemical structures of nanoparticles (graphene-like [313] and SWCNT-like [324]) consisting of C, BN, Si, Ge, MoS_2_ on the structure of the solvation layer of reline. It was found that for graphene-like nanoparticles, a stable near-surface layer of DES components is formed. This layer is dominated by urea molecules due to the formation of H-bonds and there is a lack of choline and Cl^−^ ions. A change in the radius of the SWCNT-like nanotubes did not affect the ability of the nanoparticles to undergo solvation in the DES solution. It was shown that the liquid-like structure of the DES solution is practically unchanged even near the surface of the SWCNT-like nanoparticles. Elbourne et al. [317] studied the emerging patterns of DES molecules on graphene surfaces. The orientation of molecules depending on their distance from the surface was studied. It was suggested that the formation of a near-surface DES layer implies the emergence of a separate nanostructure of the adsorbed layer from the DES components. The appearance of such a structure is apparently caused by a balance between the “surface–liquid” and “liquid–liquid” interactions, as well as the limiting effect of the solid surface at the interface.

### 3.6. Biomolecules in DES

As discussed in Introduction, DESs have a wide range of applications in pharmaceuticals as solvents, active pharmaceutical ingredients [13] and cryoprotective agents [325]. For example, it is well-known that in water solution, urea forms H-bonds with proteins breaking intramolecular protein contacts and causing denaturation [326,327,328]. A surprising and counterintuitive fact is the observed stabilization of protein structure in the DES based on urea and ChCl reported by Gorke et al. [329]. To explain this, Monhemi et al. compared the results of an MD simulation of *Candida antarctica* lipase B in reline and in urea solution [330]. It was shown that ChCl limits the diffusion of urea molecules to the protein core. Moreover, reline components form H-bonds with residues of the enzyme leading to greater enzyme stability, instead of its denaturation. This problem was further investigated by Chakrabarti’s group [331,332]. In their first work, they analyzed the effect of the ammonium salt on stabilization of DESs based on urea [331]. Comparison of peptide structures in DESs based on two different ammonium salts (ChCl and triethylammonium acetate chlorides) in different compositions (relative proportions of 1:2 molar ratio and 1:5 molar ratio) showed that reducing the concentration of ammonium salt leads to a destabilization of the protein structure. However, in case of ChCl, the protein remains more stable than in case of triethylammonium acetate. In their next work [332], Chakrabarti et al. revealed the molecular mechanisms of protein stabilization in a series of simulations of the HP-36 peptide, fully confirming the results of Monhemi et al. [330]. In addition, Pal et al. demonstrated the stabilization effect of glyceline on the protein [333]. MD simulations in this case revealed stabilizing H-bonds between the glycerol and protein residues. These H-bonds make the protein more rigid and structurally stable at high temperatures.

The same group recently studied the effect of reline on the structure of nucleic acids (Thrombin-Binding G-quadruplex Aptamer (TBA)) [334,335]. In their first work, Pal et al. demonstrated that an increase in reline concentration in water solution decreases the flexibility of TBA [334] suggesting that reline is a good choice for nucleic acid storage. In another publication, Pal et al. explored the temperature-mediated conformational dynamics of c-kit oncogene promoter G-quadruplex DNA in reline [333]. The authors demonstrated increased thermal stability of the DNA structure similar to what was observed for proteins [333] and revealed the molecular interactions responsible for these phenomena.

A series of publications from Aparicio’s group [149,150,151,336] deserves special attention. In their work on lidocaine solubility, they predicted that the solubility of lidocaine in two different DESs (ChCl + lactic acid, β-alanine + lactic acid) is several orders of magnitude larger than in water and revealed the interactions responsible for the solvation [55]. In the follow-up work, they studied the solubility of lidocaine in three newly designed DESs based on arginine and three different organic acids (glutamic acid, oxalic acid, tartaric acid) [150]. They showed that long-lived H-bonds between lidocaine and both arginine and organic acids are the main reason for the high solubility of lidocaine and demonstrated how the solubility of medicinal compounds in a DES can be controlled by the selection of suitable HBDs and HBAs. In their most recent work [151], Aparicio’s group investigated the behavior of two β-lactam antibiotics (piperacillin and ampicillin) in the same arginine DESs [150] and determined its structure and the main interactions between DESs and the antibiotics.

Another important work of Aparicio’s group focused on the investigation of cytotoxicity of different DESs by the simulation of lipid biomembranes in eleven DESs based on ChCl and different HBDs [336]. Atilhan et al. showed that the free HBDs of the DESs are inserted in the bilayer [336]. The authors showed that the insertion of HBDs initiates bilayer disruption. These results predict high cytotoxicity of the concentrated solutions of DESs. However, the number of HBDs inserted in the bilayer is strictly dependent on the hydrophobicity of the HBD. Thus, this work demonstrates the ability to regulate cytotoxicity by varying the HBD type in the DES [336].

It can be concluded that DESs are prospective solvents for pharmaceutical applications. QC and MD techniques have demonstrated their ability to resolve the intermolecular interactions and how DESs can stabilize the structures of biomolecules, such as DNA and proteins. DESs’ ability to dissolve antibiotics, together with their low toxicity, makes them promising solvents in pharmacology.

### 3.7. Biomass Pretreatment by DES

Owing to their low cost and toxicity, biodegradability, high thermal, and chemical stability, DESs have emerged as promising solvents for the pretreatment of biomass, i.e., by-products from plants, animals, and micro-organisms [337]. DESs could be used for the dissolution and separation of biomass, the exaction of useful chemicals from biomass, as well as for biomass conversion [337]. It is highly important for the optimization of biomass pretreatment to understand the molecular mechanisms responsible for the process. In this regard, computational methods have proved to be irreplaceable. Below we give typical examples of such studies.

**Figure 5:**
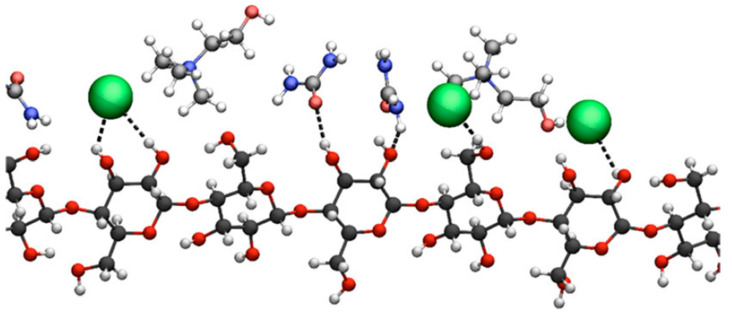
Snapshot illustrating the typical interactions of cellulose with Cl^−^ ions and urea molecules. Gray dotted lines show H-bonds. Red balls represent oxygen atoms, blue balls—nitrogen atoms, white balls—hydrogen atoms, grey balls—carbon atoms and green balls—Cl^−^ ions. Reprinted by permission from: Springer Nature, Cellulose, ref. [125], Copyright 2020.

Mohan et al. [338] studied the dissolution of glucose in DESs based on tetrabutylammonium bromide using DFT calculations and MD simulations. These techniques allowed the analysis of the glucose-DES interactions. It was shown that the anion of the hydrogen bond acceptor and the HBD molecules form hydrogen bonds with glucose and thus govern the dissolution of glucose. Similar conclusion was made by Smirnov et al. [125] in their simulation of nanocrystalline cellulose in reline. In particular, it was revealed that the formation of H-bonds between the cellulose hydroxyl groups, the urea CO group and the Cl^−^ ions are the key for dissolution, see Figure 5. The importance of H-bonds for cellulose dissolution was also demonstrated in the case of 1,8-diazabicyclo[5.4.0]undec-7-enium based DES [339]. In their study [339], Fu et al. utilized DFT calculations to describe interactions in systems consisting of cellobiose and DES molecules. It was established that both the HBA and HBD in the DES interact with cellulose via H-bonds. It was also proposed that this interaction could destroy the H-bond network of cellulose chains, thereby promoting the dissolution process. ILs dissolve cellulose in a similar fashion [340] with anions playing a major role in cellulose dissolution.

It is worth mentioning that the cellulose-based products obtained from biomass could be used to develop novel DES-containing materials. For example, Smith et al. [127] reported experimental and simulation results on bacterial cellulose gels containing DES glyceline. Both X-ray diffraction analyses and MD simulations confirmed that the DESs has almost no effect on the crystalline structure of cellulose. Moreover, MD simulations allowed the authors to explain the increased diffusion rates for DES components in gels, which was verified by nuclear magnetic resonance; it was suggested that faster diffusion stems from the migration of chloride from the bulk to the cellulose surface.

Some studies [199,341] have focused on the dissolution of lignin from biomass. Muley et al. [341] investigated lignin tetramers immersed in a DES consisting of oxalic acid and ChCl under external electric fields using MD simulations. They showed that an electric field could lengthen certain lignin bonds and, therefore, increase their probability of breaking, thus contributing to biomass deconstruction. Xu et al. [199] performed DFT calculations together with principal component analyses to evaluate factors influencing lignin removal when using ChCl-based DESs. It was established that the hydrophilic ability, polarity, acidity, and ability to form H-bonds have a significant effect on the pretreatment process.

Recently, an effective procedure has been developed for β-carotene extraction from pumpkins using DESs based on fatty acids [197]. To this end, the RSM and ANN approaches were used to optimize the extraction process. Namely, they allowed selecting the temperature, ultrasound power, and solvent to solid ratio to reach the highest yield of β-carotene from pumpkin.

## 4. Future Directions

The field of computational research of DES systems is rapidly evolving. During the past five years, a number of fundamental questions about the relation between DESs’ structure and properties have been answered with the aid of computer simulations. It is now well understood that the nature of the eutectic phenomena of DESs is directly related to the H-bond network and its subtleties. Understanding the role of each of components in classic DESs (such as reline, ethaline, or glyceline), and the interactions between them, allows for obtaining the desired structural and transport properties. For example, increasing the number of donor sites of an HBD not only should change the eutectic composition but also increase the stability of the H-bond network, and, as a result, raise viscosity. An increase in the hydrophobic part of the cation should lead to the appearance of structural heterogeneities. However, the development of new DESs with improved properties sets new tasks for computational chemistry. For example, the development of ternary DESs has great potential to enhance their properties. At the same time, increasing the number of DES components significantly complicates the prediction of the properties and emphasizes the role of computational research as an indispensable step in DES development.

The development of DESs with application-specific optimal properties is a large and active field in computational research. Most of the applications are related to the interactions of DESs with various compounds and mediums. However, in some research areas, such as solubility studies with biomolecules and molecular mechanisms of the interactions between DESs and nanoparticles, there are currently only a few investigations. Although these provide much needed information about studied systems, the low number of these publications does not allow obtaining comprehensive fundamental knowledge about the molecular processes. Moreover, the appearance of new DES applications requires knowledge from computational chemistry. For example, the recent developments of polarizable DESs for 3D printing open questions about how to control DESs’ viscosity and the complex mixtures based on them [12]. These questions can be answered using computer simulation techniques. It is important to note that DES models and approaches are currently imperfect and, in some cases, cannot provide quantitatively correct information. As can be seen from this review, work is underway to develop new approaches.

The vast growth of experimental and computational works devoted to DES publications provides a significant increase in the amount of new data. This creates a fertile field for ML techniques that should kickstart a new stage of the DES evolution.

In this review, it has been discussed in depth how particle simulations at atomistic and quantum levels provide excellent means for detailed studies of complex systems. However, these systems can only be simulated up-to nanoscale spatial lengths and nanosecond time scales. To study DESs on a larger scale, which would be desirable for industrial applications for instance, simulation techniques that can model systems on the meso- (intermediate) or macroscale might be better suited. In particular, it would be desirable to capture the microstructure evolution of a DES, for example, near its eutectic point. Furthermore, meso- and macroscale modeling can also provide information about the behavior of parameters such as chemical potential, surface tension and viscosity, necessary for advancing our understanding of DESs [37].

At a high level of coarse-graining [164], phase-field modeling (PFM) is a very powerful mathematical and computational framework for microstructure simulations [342]. In PFM, instead of particles, a system is represented using one or more continuous variables called order parameters and the corresponding set of partial differential equations can be determined from a free energy functional or even derived phenomenologically. The order parameters are typically based on the symmetries of the system around a phase transition, e.g., the eutectic point, and represent the phase as a field of values (hence the name phase-field) at a mesoscopic scale. The appeal of PFM comes from its ability to capture large-scale behavior. Additionally, and very importantly, the interfaces between the bulk regions and the complex dynamics emerge naturally from the construction of a PFM [343]. The PFM approach has been successful in a wide variety of application, such as directional solidification and dendritic growth [344,345,346], formation of polycrystalline structures [347,348], cardiac electric signals [349,350], and crystal growth [349,351] and electrochemical effects [352]. On the computational side, PFM is highly amenable to large-scale parallelizable simulations and there are a number of software packages available to simulate various phase-field models based on both finite element and finite difference methods that usually employ some level of parallelization [353,354,355,356,357,358].

Although, currently, there are no direct applications of PFM to DESs, it is a potential new approach and has been used in modeling the formation and behavior of eutectic materials. The application of PFM to eutectics initially began with describing isothermal phase transitions in binary alloys [359]. This work was extended to include eutectic growth by Elder et al. [360] and by Karma [361]. The disadvantage of the initial attempts of formulating a model was that the pure substances had an infinitely high melting temperature. There were further extensions to mitigate this issue, as well as to include non-isothermal eutectic systems [362,363]. Recent work on PFM of eutectics includes studies of directional solidification of ternary eutectics by Hötzer et al., who base their PFM on multicomponent alloy solidification [364,365,366,367] and simulate a ternary eutectic at the three phase invariant point to study microstructure formation. Additionally, phase-field simulations were performed for the ternary eutectic Al–Ag–Cu by Steinmetz et al., who found good agreement of directional solidification patterns with experimental results [368].

One step to applying PFM to DESs is understanding the phase behavior of such systems. Some work has been performed on this topic; a study by Kollau et al. showed that one of the difficulties in adequately describing the phase behavior arises because typical DES components often have different sizes and shapes, thus, an ideal entropy of mixing does not appropriately represent DESs. They found that significant differences in the phase behaviors of DESs come from the choice of entropy expressions and molar volumes [369].

It is important to note that despite all of our efforts, not all publications devoted to DES simulations were included in the review due to their high number. To help the reader and to put the studies that we may have missed, and new studies that become published, in a perspective, we present systemized information about the publications in Table A1, Table A2 and Table A3 in Appendix A with brief information about every publication.

## Figures and Tables

**Table 1:** Types of DESs, their compositions, and examples.

Type	Composition	Example
Type I	Organic and metal salts	Choline chloride + Metal halide (SnCl_2_, ZnCl_2_, etc.)
Type II	Organic and metal salt hydrate	Choline chloride + Metal salt hydrate (CrCl_3_∙6H_2_O, etc.)
Type III	Organic salt and H-bond donor	Choline chloride + organic compound (urea, carboxylic acids, alcohols, etc.)
Type IV	Metal salt and H-bond donor	Metal halide (ZnCl_2_) + organic compound (urea, carboxylic acids, alcohols, etc.)
Type V	Non-ionic DESs. Hydrogen Bond Acceptor and Hydrogen Bond Donor	Thymol + menthol

## Appendix A

**Table A1:** Atomistic simulations of DES consisting systems, force field used, and the topic of the publication. It should be noted that the partial charges may vary greatly in the publications with the same force field due to variations of approaches for their calculations. We refer to current publications for the details.

No	DES	FF/Corrections	Topic of the Publication	Reference
1	1-ethyl-3-methylimidazolium chloride:Urea Different molar ratios	CL&P OPLS-AA	Investigation of DES structure	[231]
2	1-octyl-3-methylimidazolium: Tetrafluoroborate 1-ethyl-3-methylimidazolium:Tetrafluoroborate Different molar ratios	CL&P	Investigation of DES structure	[370]
3	Acetamide:LiBr (3.5:1)	CHARMM 27 only/no corrections	Support to experiment	[253]
4	Acetamide:LiClO_4_ (3.5:1)	CHARMM 27 + separate parameters for ions	Mechanisms of DES components motion	[249]
5	Acetamide:LiClO_4_ (3.5:1)	CHARMM 27 + separate parameters for ions	Mechanism of Acetamide in Deep Eutectic Solvents	[250]
6	Acetamide:LiClO_4_ (3.5:1) Acetamide:LiNO_3_ (3.5:1) Acetamide:LiBr (3.5:1)	CHARMM 27 + separate parameters for ions	Orientation jumps of acetamide molecules	[253]
7	Acetamide:LiClO_4_ (4.1:1) Acetamide:LiNO_3_ (3.5:1) Acetamide:LiBr (3.5:1)	CHARMM 27 + separate parameters for ions	Support to experiment	[252]
8	Acetamide:LiNO_3_ (3.5:1)	CHARMM 27 + separate parameters for ions	Mechanisms of DES components motion	[248]
9	Acetamide:Urea (1.5:1) Acetamide:Urea (2.3:1)	CHARMM + GROMOS96/no corrections	Temperature dependent relaxation dynamics, particle motion characteristics, and heterogeneity aspects of deep eutectic solvents	[254]
10	Acetamide:Urea (2.3:1)	CHARMM + GROMOS96/no corrections	Structural H-bond relaxation	[255]
11	Acetamide:Urea (2.3:1)	OPLS-DES/no corrections	Heterogeneity of reorientational relaxation and translational dynamics	[256]
12	Arginine:Glutamic acid (1:1) Arginine:Oxalic acid (1:1)Arginine:Tartaric acid (1:1)	No name of FF. Parameters are given	Lidocaine in DES	[150]
13	Arginine:Glutamic acid (1:1) Arginine:Oxalic acid (1:1)Arginine:Tartaric acid (1:1)	No name of FF. Parameters are given	Antibiotics in DES	[151]
14	Benzene-1,4-diol:Urea Different molar ratios	AMBER99	Investigation of DES structure and dynamics and water effect on it	[371]
15	Betaine:Lactic acid (1:1)	No name of FF. Parameters are given	Investigation of DES structure	[153]
16	Betaine Monohydrate:Glycerol Different molar ratios	GAFF/no corrections	Extraction of palmitic acid by DES	[372]
17	Betaine Monohydrate:Glycerol (1:2)	GAFF/no corrections	Investigation of DES structure	[373]
18	Bis(trifluoromethanesulfonyl)imide:Methanesulfonamide Bis(trifluoromethanesulfonyl)imide:Dimethylmethanesulfonamide Different molar ratios	OPLS-AA/no corrections	Characterization of the transport properties of binary DESs	[374]
19	Caprolactam:Tetrabutylammonium bromide (1:1) Caprolactam:Tetrabutylammonium bromide (1:1) ChCl:Urea (1:2) Methyltriphenylphosphonium bromide:Monoethanolamine (1:6)	Gromos54a7, the optimized forcefield parameters were obtained from the Automated Topology Builder (ATB) database	Natural Gas Desulfurization using DES	[375]
20	Ceineole:Succinic acid (1:1) Ceineole:Malic acid (1:1) Ceineole:Lactic acid (1:1)	No name of FF. Parameters are given	Investigation of DES structure	[154]
21	ChCl:Ethylene glycol (1:4)	OPLS-AA/no corrections	DES for gas separation	[276]
22	ChCl:1,2-ethanediol (1:2) ChCl:1,3-propanediol (1:3) ChCl:1,4-butanediol (1:3)	OPLS and GAFF/no corrections	Dependence of Solvation Dynamics in Alcohol-Based DES	[376]
23	ChCl:1,2-ethanediol ChCl:1,3-propanediol ChCl:1,4-butanediol ChCl:1,5-pentanediol	Combination of OPLS-AA and Amber parameters	Investigation of DES structure	[377]
24	ChCl:Acetyl salicylic acid (1:1)	GAFF/no corrections	Investigation of DES structure	[222]
25	ChCl:Citric acid (1:1) ChCl:Fructose (1:1) ChCl:Malic acid (1:1) ChCl:Lactic acid (1:1)	No name of FF.Parameters are given	Gas Solubility and Rheological Behavior of Natural DES	[54]
26	ChCl:Ethylene glycol Different molar ratios	OPLS-AA/no corrections	DES for CO_2_ separation	[274]
27	ChCl:Ethylene glycol (1:1) ChCl:Ethylene glycol (1:2)	OPLS-AA CL&P/charges scaling for ions is 0.8	Water effect on DES structure	[294]
28	ChCl:Ethylene glycol (1:2)	GAFF/ charges scaling for ions is 0.9	CO_2_ uptake by a DES in slit nanopores	[311]
29	ChCl:Ethylene glycol (1:2)	OPLS/no corrections	DES at a solid electrode	[315]
30	ChCl:Ethylene glycol (1:2)	INTERFACE and CGenFF	DES at solid interfaces	[317]
31	ChCl:Ethylene glycol (1:2)	GAFF/charges scaling for ions is ±0.9	Investigation of DES structure	[161]
32	ChCl:Ethylene glycol (1:2)	CHARMM General Force Field/no corrections	Investigation of DES structure	[213]
33	ChCl:Ethylene glycol (1:2)	FF developed by authors (0.8 FFM3) based on OPLS	Investigation of DES structure	[214]
34	ChCl:Ethylene glycol (1:2)	CL&Pol	Force field validation	[137]
35	ChCl:Ethylene glycol (1:2)	CL&PolCL&P	Force field validation	[140]
36	ChCl:Ethylene glycol (1:2)	OPLS-AA/no corrections	Water effect on DES physicochemical properties	[302]
37	ChCl:Ethylene glycol (1:2)	GAFF/no corrections	Solvatochromic parameters, and preferential solvation in aqueous solutions of DES and its components	[293]
38	ChCl:Ethylene glycol (1:2)	OPLS/no corrections	CO_2_ absorption in DES	[158]
39	ChCl:Ethylene glycol (1:2) ChCl:Glycerol (1:2) ChCl:Malonic acid (1:2)	GAFF/charge scaling for ions is 0.9	Investigation of DES structure	[124]
40	ChCl:Ethylene glycol (1:2) ChCl:Glycerol (1:2) ChCl:Urea (1:2)	GAFF/no corrections	Solvation dynamics of an ionic probe in DES	[157]
41	ChCl:Ethylene glycol (1:2) ChCl:Glycerol (1:2) ChCl:Urea (1:2)	GAFF/no corrections	Investigation of separation mechanism of methanol hexane mixtures	[285]
42	ChCl:Ethylene glycol (1:2) ChCl:Levulinic acid (1:2)	GAFF/charge scaling for ions is 0.9 for systems with ethylene glycol 0.8—for systems with levulinic acid	Fluorinated refrigerants in DESs	[120]
43	ChCl:Ethylene glycol (1:2) ChCl:Levulinic acid (1:2)	ChCl and Ethylene glycol–GAFF Levulinic acid-HF/6–31G* + AMBER or DFT for optimized clusters	DES for gas separation	[275]
44	ChCl:Ethylene glycol (1:2) ChCl:Propylene glycol (1:2) ChCl:1,3-propanediol (1:2) ChCl:Glycerol (1:2)	FF developed by authors (0.74 FFM16) based on + OPLS	Investigation of DES structure	[217]
45	ChCl:Ethylene glycol (1:3) ChCl:Glycerol (1:3)	COMPASS	Silica nanoparticles in DES	[378]
46	ChCl:Ethylene urea (1:2) ChCl:Thiourea (1:2) ChCl:Ethylene glycol (1:2) ChCl:Glycerol (1:2)	“UNIVERSAL” force field Forcite and Blends Module Materials studio package	Desulfurization mechanism by the DES solvents	[280]
47	ChCl:Glucose (1:3, 1:1, 3:1)	CHARMM 36/no corrections	Investigation of DES structure	[379]
48	ChCl:Glycerol	OPLS-AA/no corrections	Gels based on DES and cellulose	[127]
49	ChCl:Glycerol:Resorcinol (1:5:3)	AMBER Parameters are given	Investigation of DES structure	[246]
50	ChCl:Glycerol (1:2)	CHARMM 36/no corrections	Water effect on DES structure	[287]
51	ChCl:Glycerol (1:1)	Gromos54a7/no corrections	Effects of a cholinium based DES on function and structure of versatile peroxidase	[380]
52	ChCl:Glycerol (1:2, 1:3)	GAFF/no corrections	Investigation of DES structure	[215]
53	ChCl:Glycerol (1:2)	GAFF/charge scaling is 0.9	Protein in DES	[333]
54	ChCl:Glycerol (1:2)	GAFF/no corrections	solvation of enzyme in DES	[381]
55	ChCl:Glycerol (1:2) ChCl:Ethylene glycol (1:2)	GAFF OPLS-DES CGenFF no corrections	Validation of force field for mixtures of DES and water and interaction DES with enzyme	[382]
56	ChCl:Glycerol (1:2)	GAFF/charge scaling for ions is 0.9	Effect of water on thermophysical properties DES	[304]
57	ChCl:Lactic acid (1:1) β-alanine:Lactic acid (1:1)	No name of FF. Parameters are given	Lidocaine in DES	[55]
58	ChCl:Lactic acid (1:9)	GAFF/no corrections	Lignin dissolution behaviors of DES	[383]
59	ChCl:Levulinic acid (1:2)	No name of FF. Parameters are given	DES on the metal surface	[318]
60	ChCl:Levulinic acid (1:2)	No name of FF. Parameters are given	DES for CO_2_ capture	[52]
61	ChCl:Malonic acid ChCl:Oxalic acid ChCl:Succinic acid ChCl:Fumaric acid Different molar ratios	GAFF/no corrections	Investigation of DES structure	[221]
62	ChCl:Malonic acid (1:1, 1:2)	GAFF/no corrections	Separation of nitrogen-containing aromatics by DES	[384]
63	ChCl:Oxalic acid (1:1)	CHARMM 36/no corrections	Biomass separation	[341]
64	ChCl:Phenyl propionic acid (1:2)	CHARMM 36/charges scaling is 0.8.	Investigation of DES structure	[224]
65	ChCl:Phenyl propionic acid Different molar ratios	CHARMM 36/no corrections	Investigation of DES structure	[223]
66	ChCl:Phenylacetic acid (1:2)	No name of FF. Parameters are given	CO_2_ absorption with DES	[53]
67	ChCl:Propylene glycol (1:2) ChCl:Ethylene glycol (1:2)	FF developed by authors (0.74 FFM16) based on OPLS	Investigation of DES structure	[216]
68	ChCl:Sesamol (1:3)	OPLS-AA/no corrections	Water effect on DES structure	[296]
69	ChCl:Urea (1:2)	No name of FF. Parameters are given	Investigation of enhanced oil recovery by DES	[385]
70	ChCl:Urea Different molar ratios	CL&P/charge scaling for ions is 0.9.	Investigation of DES structure	[214]
71	ChCl:Urea Different molar ratios	GAFF/charge scaling for ions is 0.8	Investigation of DES structure	[123]
72	ChCl:Urea (1:2)	GROMOS 96/no corrections	Protein in DES	[330]
73	ChCl:Urea (1:2)	GAFF/charge scaling is 0.8	DNA in DES	[335]
74	ChCl:Urea (1:2)	OPLS-AA/no corrections	Protein in DES	[332]
75	ChCl:Urea (1:2)	GAFF/charge scaling is 0.8	DNA in DES	[334]
76	ChCl:Urea (1:2)	CHARMM 22/no corrections	DES near graphene	[322]
77	ChCl:Urea (1:2)	No name of FF. Parameters are given	DES at 2D nanomaterial interfaces	[313]
78	ChCl:Urea (1:2)	No name of FF. Parameters are given	DES with different nanotubes	[324]
79	ChCl:Urea (1:2)	Combine force field based on CGenFF and CHARMM 36	DES nanodroplet at carbon material	[323]
80	ChCl:Urea (1:2)	GAFF/ charges scaling is 0.9 for the ions	DES droplets on ionic substrates	[320]
81	ChCl:Urea (1:2)	OPLS-AA/no corrections	Dissolution of cellulose in DES	[21]
82	ChCl:Urea (1:2)	q4-MD force field/no corrections	Molecular encapsulation	[386]
83	ChCl:Urea (1:2)	Monte Carlo/Empirical potential structure refinement (EPSR)	Investigation of DES structure	[387]
84	ChCl:Urea (1:2)	OPLS-AA/no corrections	Separation of uranyl Ions	[388]
85	ChCl:Urea (1:2)	Various force-fields	Force fields comparison	[228]
86	ChCl:Urea (1:2)	OPLS-AA/no corrections	Flow resistance of DES confined in ionic model nanoslits	[389]
87	ChCl:Urea (1:2)	CHARMM 36/no corrections	Conformation and Stability of Lysozyme in DES/water mixtures	[291]
88	ChCl:Urea (1:2)	CHARMM 36/no corrections	Stability and activity of lipase in DES/water mixtures	[305]
89	ChCl:Urea (1:2)	OPLS-AA/no corrections	Water effect on DES structure	[295]
90	ChCl:Urea (1:2)	CHARMM 36/no corrections	Water effect on DES structure	[286]
91	ChCl:Urea (1:2)	CHARMM 36/charge scaling is 0.89	Water effect on DES structure	[292]
92	ChCl:Urea (1:2)	OPLS-AA/no corrections	Effect of DES on the water structure	[300]
93	ChCl:Urea (1:2)	Merck Molecular Force Field (MMFF)	Water effect on DES thermo-physical properties	[36]
94	ChCl:Urea (1:2)	Gromos54a7/no corrections	Intermolecular interactions between DES and dimethylsulfoxide	[390]
95	ChCl:Urea (1:2) Butiyltrimetylammonium chloride:Urea (1:2)	Lopes-Padua and OPLS-AA/no corrections	Investigation of DES structure	[211]
96	ChCl:Urea (1:2) ChCl:Ethylene glycol (1:2)	GAFF based FF/charge scaling for ions is 0.9 for systems with urea 0.8—for systems with ethylene glycol	Water effect on DES properties	[298]
97	ChCl:Urea (1:2) ChCl:Ethylene glycol (1:2)	Merck Molecular Force Field (MMFF)	Water effect on DES properties	[303]
98	ChCl:Urea (1:2) ChCl:Ethylene Glycol (1:2)	No name of FF. Aspen plus simulator	CO_2_ removal from shale gas by DESs	[273]
99	ChCl:Urea (1:2) ChCl:Ethylene glycol (1:2) ChCl:Glycerol (1:2)	GAFF based FF/charge scaling for ions is 0.9 for systems with urea 0.8—for systems with ethylene glycol or glycerol	DES for absorption refrigeration systems	[391]
100	ChCl:Urea (1:2) ChCl:Ethylene glycol (1:2) ChCl:Glycerol (1:2)	GAFF based FF/charge scaling for ions is 0.9 for systems with urea 0.8—for systems with ethylene glycol or glycerol	Water effect on DES thermal conductivity	[299]
101	ChCl:Urea (1:2) ChCl:Ethylene glycol (1:2) ChCl:Glycerol (1:2)	Gromos54a7/no corrections	Water effect on DES structure	[392]
102	ChCl:Urea (1:2) ChCl:Ethylene glycol (1:2) ChCl:Glycerol (1:2)	GAFF/no corrections	Solvatochromic properties and ion solvation structure in DESs	[393]
103	ChCl:Urea (1:2) ChCl:Ethylene glycol (1:2) ChCl:Glycerol (1:2)	GAFF/no corrections	Solvatochromic behavior of dimethyl sulfoxide with DESs	[394]
104	ChCl:Urea (1:2) ChCl:Ethylene glycol (1:2) ChCl:Glycerol (1:2) and their mixtures	No name of FF. Parameters are given	DESs on 2D materials	[321]
105	ChCl:Urea (1:2) ChCl:Ethylene glycol (1:2) ChCl:Glycerol (1:2) ChCl:Malonic acid (1:1) ChCl:Oxalic acid (1:1)	OPLS, GAFF/scaling of 1–4 intramolecular interaction energies	Hildebrand and Hansen solubility parameters of DESs	[395]
106	ChCl:Urea (1:2) ChCl:Ethylene glycol (1:2) ChCl:Glycerol (1:2) ChCl:Oxalic acid (1:1) ChCl:Malonic acid (1:1) ChCl:Glutaric acid (1:1) ChCl:Malic acid (1:1) ChCl:Citric acid (1:1) ChCl:Levulinic acid (1:2) ChCl:Phenyl acetic acid (1:2) ChCl:Acetamide (1:2)	Combine force field based on CGenFF and CHARMM 36	Lipid membrane in DES	[336]
107	ChCl:Urea (1:2) ChCl:Ethylene glycol (1:2) ChCl:Glycerol (1:2) ChCl:Propylene glycol (1:2)	GAFF/no corrections	extraction mechanism of 1-butanol separation from alkanol azeotropic system using choline-based DES	[284]
108	ChCl:Urea (1:2) ChCl:Ethylene glycol (1:2) ChCl:Levulinic acid (1:2) Betaine:Levulinic acid (1:2)	Modified AMBER force field	Mercury Capture from Petroleum using DES	[159]
109	ChCl:Urea (1:2) ChCl:Ethylene Glycole (1:2) ChCl:Glycerol (1:2) ChCl:2-aminoethan-1-ol (1:6) Allyltriphenyl phosphonium bromid:Ethylene glycol (1:4) Allyltriphenyl phosphonium bromid:Triethylene Glycol (1:4) Allyltriphenyl phosphonium bromid:Levulinic acid (1:4)	No name of FF. Forcite module, from Materials Studio software	Molecular interaction between the different DESs and CO_2_	[277]
110	ChCl:Urea (1:2) ChCl:Glycerol (1:2) ChCl:Levulinic acid (1:2) ChCl:Malonic acid (1:1) ChCl:Phenylacetic acid (1:2)	No name of FF. Parameters are given Charges are calculated for clusters.	Investigation of DES structure	[396]
111	ChCl:Urea (1:2) ChCl:Glycerol (1:2) ChCl:Malonic acid (1:1)	No name of FF. MDynaMix v.5.2 molecular modeling software	Mechanisms of acid gases capture in DES	[268]
112	ChCl:Urea (1:2) ChCl:Glycolic acid (1:1)	OPLS/charge scaling is 0.8	Stability and activity of alcohol dehydrogenase in DES/water mixtures	[288]
113	ChCl:Urea (1:2) ChCl:Thiourea (1:2) ChCl:Methyl urea (1:2) ChCl:Dimethyl urea (1:2) ChCl:1,1-dimethylurea (1:2)ChCl:*N*,*N*′-ethylene urea (1:2)	GAFF/charges scaling is 0.9 for urea and 0.8 for ChCl	Investigation of DES structure	[118]
114	ChCl:Urea (1:2) ChF:Urea (1:2) ChNO_3_:Urea (1:2) Ch acetate:Urea (1:2)	CL&P	Investigation of DES structure	[230]
115	ChCl: Water	ChCl: LJ parameters (AMBER) + partial charges (DFT) Water: TIP3P	Water structuring in DES	[397]
116	ChCl:Water (1:3.3)	OPLS-AA/no corrections	Investigation of DES structure	[398]
117	ChCl:Water	OPLS-AA/no corrections	Effect of ChCl on water structure	[301]
118	ChCl derivatives consisted of a series of elongated alkyl side chains and elongated alcohol side chains [Ch]^+^, [C_4_Ch]^+^, [C_6_Ch]^+^, [C_8_Ch]^+^, [(C_4_)_3_Ch]^+^, [ChC_4_OH]^+^, [ChC_6_OH]^+^, [ChC_8_OH]^+^, [ChC_10_OH]^+^, [ChC_12_OH]^+^) HBD:Ethylene glycol Molar ratio 1:4	CL&P	Investigation of DES structure	[239]
119	Choline chloride:Trifluoroacetamide (1:2.5) Chlorocholine chloride:Trifluoroacetamide (1:2.5) Tetrametilammonium chloride:Trifluoroacetamide (1:2.5) Tetraethylammonium chloride:Trifluoroacetamide (1:2.5) Benziltriethylammoniun chloride:Trifluoroacetamide (1:2.5)	GAFF/no corrections	Investigation of DES structure	[240]
120	Choline iodide:Glycerol (1:3)	GAFF/ charges scaling is 0.9 for for the ions	DES in nanopores	[309]
121	Decanoic acid:Menthol (1:1, 1:2) Decanoic acid:Lidocain (2:1) Menthol:Lidocain (2:1) Thymol:Lidocaine (1:1, 2:1) Thymol:Menthol (1:1, 2:1)	OPLS-AA/M Parameters are given	Investigation of DES structure	[238]
122	Menthol:Lauric acid 2:1 Menthol:Decanoic acid 1:1	GAFF/no corrections	Investigation of DES structure	[19]
123	dl-menthol:Hexanoic acid (1:1) dl-menthol:Octanoic acid (1:1) dl-menthol:Decanoic acid (1:1)	CHARMM 36/no corrections	Dynamics of hydrogen-bonding and translational dynamics and their dependence on acid tail length	[399]
124	dl-menthol based DESs HBDs: Acetic acid, Butanoic acid, Hexanoic acid, Octanoic acid, Decanoic acid, Dodecanoic acid, Pyruvic acid, Levulinic acid Tetrabutylammonium chloride based DESs HBDs: Acetic acid, Octanoic acid	GAFF/no corrections	Water Stability of Hydrophobic DES	[219]
125	Ferric chloride:Tetrabutylphosphonium bromide Different molar ratios	Gromos54A7	Investigation of DES structure	[400]
126	L-Arginine:Glutamic acid (1:1) L-Arginine:Oxalic acid (1:1) L-Arginine:Tartaric acid (1:1)	No name of FF. Parameters are given	Nitric oxide solubility in DES	[152]
127	l-Menthol:Acetic acid (1:1)	AMBER 14	Structure Elucidation of DES	[155]
128	LiBr:Acetamide (4.5:1) LiNO_3_:Acetamide (4.5:1) LiClO_4_:Acetamide (4.5:1)	OPLS-UA	Investigation of DES structure	[244]
129	LiBr:Acetamide (4.5:1) LiNO_3_:Acetamide (4.5:1) LiClO_4_:Acetamide (4.5:1)	CHARMM 22 for nucleic acids	Investigation of DES structure	[243]
130	LiCl and LiTFSI based DESs HBDs: Urea, Acetamide:*N*,*N*_0_-dimethylpropyleneurea, 2-imidazolidinone, TetramethylureaMolar ratio 1:5	COMPASS II	Investigation of DES structure	[245]
131	LiClO_4_:Acetamide (1:3.5)	CHARMM 27/no corrections	Water effect on DES properties	[401]
132	LiClO_4_:Acetamide (1:5.2) LiClO_4_:Propion amid (1:5.2)	CHARMM 22/no corrections	Investigation of DES structure	[242]
133	Litium bis-(trifluoromethanesulfonyl)-imide: Urea Different molar ratios	OPLS-AA/no corrections	Investigation of DES structure	[402]
134	Litium bis-(trifluoromethanesulfonyl)-imide: Urea Different molar ratios	OPLS-AA/no corrections	Investigation of DES structure	[403]
135	Methyltriphenylphosphonium bromide:Ethylene glycol (1:4)	GAFF/no corrections	Extraction of benzene from hydrocarbon mixture using a phosphonium based DES	[282]
136	Methyltriphenylphosphonium bromide:Ethylene glycol (1:4)	GAFF/no corrections	Extraction of a polyaromatic hydrocarbon from fuel oils using DES	[283]
137	Methyltriphenylphosphonium bromide:Ethyleneglycol (1:4) Methyltriphenylphosphonium bromide:Glycerol (1:4) Tetrabutylammonium bromide:Ethyleneglycol (1:4) Tetrabutylammonium bromide:Glycerol (1:4)	GAFF/no corrections	Investigation of DES structure	[232]
138	Methyltriphenylphosphonium bromide:Monoethanolamine Different molar ratios	No name of FF. Parameters are given	Investigation of DES structure	[233]
139	Monoethanolamine (MEA) and Methyltriphenylphosphonium bromide (MTPPBr) based DESs. Different molar ratios	Gromos54a7, Ions: MTPP+ (AMBER), MEA and CO_2_: ATB database	CO_2_ absorption in DES	[272]
140	Monoethanolamine hydrochloride:Methyldiethanolamine Diethanolamine hydrochloride:Methyldiethanolamine *N*-methyl diethanolamine hydrochloride:Methyldiethanolamine Different molar ratios	GAFF/no corrections	CO_2_ capture performance by DES	[404]
141	Morpholine and Morpholine based DESs. HBDs: Urea, Diethylene glycol, Carboxylic acid, Thiourea, Methanol Molar ratio 1:4	OPLS-AA/no corrections	Investigation of DES structure	[405]
142	Oleic acid:Lidocaine (1:1)	GAFF/no corrections	Phase separation property of a hydrophobic DES	[306]
143	Proline:Glycolic acid Proline:Malic acid Different molar ratios	Amber-Cornell force field	Investigation of DES structure	[229]
144	Tetraalkylammonium chloride:Decanoic acid (1:2) Cation alkylchain lengths-4, 7, 8)	GAFF/charges scaling is 0.6–1.0 LJ well-depth scaling factors	Investigation of DES structure	[237]
145	Tetrabutilamonium chloride:Ethylene glycol (1:3) Tetrabutilamonium chloride:Glycerol (1:5)	OPLS-AA/partial charges scaling for ions is 0.8	Investigation of DES structure	[406]
146	Tetrabutylammonium bromide:Formic acid (1:1)	No name of FF. Parameters are given	Oil desulfurization by DES	[149]
147	Tetrabutylammonium bromide:Imidazole (1:2) Tetrabutylammonium bromide:Ethylene glycol (1:4) Tetrabutylammonium bromide:Glycerol (1:4)	GAFF/no corrections	Dissolution of carbohydrates in DES	[338]
148	Tetrabutylammonium bromide:Sulfolane Different molar ratios	OPLS-AA and CL&P	Quantification of the total vapor pressures of DESs	[407]
149	Tetrabutylammonium chloride:Decanoic acid (1:2) Thymol:Decanoic acid (1:2) dl-menthol:Decanoic acid (2:1)	GAFF/charge scaling for ions is 0.833	Interfacial Properties of Hydrophobic DES	[307]
150	Tetrabutylammonium chloride:FeCl_3_: Polyethylene glycol (4:0.05:1)	Gromos54a7/no corrections	Investigation of DES structure	[408]
151	Tetrabutylammonium chloride:Polyethylene glycol:Ferric chloride (4:1:0.05)	Gromos54a7/no corrections	Mechanism of desulfurization by the DES	[281]
152	Tetrabutylphosphonium bromide:Phenol (1:4) Tetrabutylphosphonium bromide:Diethylene glycol (1:4) Allyltriphenylphosphonium bromide:Phenol (1:4) Allyltriphenylphosphonium bromide:Phenol (1:6)	AMBER/no corrections	CO_2_ solubility in DESs	[278]
153	Triethylammonium acetate:Urea (1:2)	OPLS-AA/no corrections	Protein in DES	[331]

**Table A2:** QM calculations of DES consisting systems, method and basis, and the topic of the publication. Corrections of method and basis are not indicated. We refer to current publications for the details.

No	DES	Method/Basis	Topic of the Publication	Reference
1	1-ethyl-3-methylimidazolium chloride: Imidazole Different molar ratios	AIMD The BLYP functional with triple-ζ valence polarization basis set and GTH pseudopotentials. CP2K/QUICKSTEP code DFT B3LYP/6-311+G(2d,2p)	DES as physical solvents for remarkable separation of H_2_S from CO_2_	[409]
2	1,8-diazabicyclo [5.4.0]undec-7-enium: methylthiourea (4:1)	DFT M06e2X/6-311++G(d,p)	Dissolution of cellulose in DES	[339]
3	24 choline chloride-based DES with molar ratio from 1:4 to 15:1	DFT B3LYP/6-311G(d,p) B3LYP/def2-SVP def2-SVP/J B3LYP/6-311G(d,p)/def2-TZVP def2-TZVP/J	Biomass separation	[119]
4	Alanine:Lactic Acid (1:1) Alanine:Malic Acid (1:1) Betaine:Lactic Acid (1:1) ChCl:Malic Acid (1:1) ChCl:Lactic Acid (1:1) ChCl:Fructose (1:1)	DFT B3LYP/6-311++G(d,p)	CO_2_ absorption by DES	[79]
5	Alanine:Lactic acid (1:1) Betaine:Lactic acid (1:1) ChCl:Lactic acid (1:1) ChCl:Malic acid (1:1) ChCl:Phenylacetic acid (1:2)	DFT B3LYP/6-311++G(d,p)	High Pressure Methane Solubility in Natural DES	[410]
6	AlCl_3_:Urea (1:1, 1.5:1)	AIMD DFT/PAW	Investigation of DES structure	[160]
7	Arginine:Glutamic acid (1:1) Arginine:Oxalic acid (1:1) Arginine:Tartaric acid (1:1)	DFT B3LYP/6-311++G(d,p)	Lidocaine in DES	[150]
8	Arginine:Glutamic acid (1:1) Arginine:Oxalic acid (1:1) Arginine:Tartaric acid (1:1)	DFT B3LYP/6-311++G(d,p)	Antibiotics in DES	[151]
9	Betain:Glycerol (1:2) Betain:dl-lactic acid (1:2) Betain:Levulinic acid (1:2)	DFT GGA/VMN-BP function in DNP 4.4 basis set	Extraction phenolic compounds from oil mixtures by DES	[95]
10	Betaine:Lactic acid (1:1)	DFT B3LIP/6-311++G(d,p)	Investigation of DES structure	[153]
11	Camphor:1-decanol Camphor:Decanoic acid Camphor:3,4-xylenol	DFT B3LYP/6-311++G(d,p)	Detoxification of feedstocks using DES	[74]
12	Caprolactam:Tetrabutylammonium bromide (1:1) Caprolactam:Tetrabutylammonium bromide (1:1) ChCl:Urea (1:2) Methyltriphenylphosphonium bromide:Monoethanolamine (1:6)	DFT B3LYP/6-311++G(d,p)	Natural Gas Desulfurization using DES	[375]
13	Ceineole:Succinic acid (1:1) Ceineole:Malic acid (1:1) Ceineole:Lactic acid (1:1)	DFT B3LIP/6-311++G(d,p)	Investigation of DES structure	[154]
14	ChCl:1,2-butanediol (1:4) ChCl:1,3-butanediol (1:4) ChCl:1,4-butanediol (1:4)	DFT DMol3 module with the generalized gradient approximation (GGA)-based Perdew-Wan (PW91) functional	The structure of DES and supercapacitor performance	[88]
15	ChCl:4-chlorophenol ChCl:4-ethylphenol ChCl:Phenol ChCl:2-methylphenol ChCl:3-methylphenol ChCl:4-methylphenol ChCl:2,6-dimethylphenol Different molar ratios	DFT M06-2x/6–31++G(d,p)	Extractive desulfurization of fuels by DES	[86]
16	ChCl:Acetyl salicylic acid (1:1)	DFT ωB97XD/6-311++G(d,p)	Investigation of DES structure	[222]
17	ChCl:Acrylic acid (1:2)	Gaussian software No name of method	Investigation of DES structure	[220]
18	ChCl:Carboxylic acid (1:2) ChCl:Formic acid (1:2)	DFT B3LYP/DNP	Investigation of DES structure	[68]
19	ChCl:Citric acid ChCl:Ethylene glycol ChCl:Fructose ChCl:Glycerol ChCl:Lactic acid ChCl:Levulinic acid ChCl:Malic acid ChCl:Phenylacetic acid 1-Butyl-3-methylimidazolium chloride:Acetamide 1-Ethyl-3-methylimidazolium chloride:Acetamide 1-Ethyl-3-methylimidazolium chloride:Ethylene glycol	DFT B3LYP/6-311++G(d,p)	Interactions Between Deep Eutectic Solvents and SO_2_	[78]
20	ChCl:d-(+)-xylose ChCl:d-(−)-ribose ChCl:d-(−)-fructose Different molar ratios	DFT B3LYP/6-311++G(d,p)	DES as absorbents for NH_3_ capture	[94]
21	ChCl:Ethylene glycol (1:2)	AIMD GPW/PBE/MOLOPT-TZVP-GTH basis set	Investigation of DES structure	[161]
22	ChCl:Ethylene glycol (1:2)	AIMD BLIP/DZVP	Investigation of DES structure	[214]
23	ChCl:Ethylene glycol (1:2)	DFT/PAW	Eutectic-mediated selective synthesis of nanocrystals	[297]
24	ChCl:Ethylene glycol (1:2)	DFT B3LYP/6-31G(d) TD-DFT CAM-B3LYP/TZVP	Solvatochromic parameters, and preferential solvation in aqueous solutions of DES and its components	[293]
25	ChCl:Ethylene glycol (1:2)	DFT B3LYP/6–311++G(d,p)	Dissolution of dimethylformamide in DES	[411]
26	ChCl:Ethylene glycol (1:2)	AIMD MOLOPT-DZVP-SR-GTH with GGA BLYP with GTH pseudopotentials to represent the core electrons	CO_2_ absorption in DES	[158]
27	ChCl:Ethylene glycol (1:2) ChCl:Glycerol (1:2) ChCl:Urea (1:2)	DFT B3LYP/6-311++G(d,p)	Solvation dynamics of an ionic probe in DES	[157]
28	ChCl:Ethylene glycol (1:2) ChCl:Malic acid (1:2) ChCl:Tartaric acid (2:1) ChCl:Oxalic acid (1:2) ChCl:Glycerol (1:2)	HF, M06, B3LYP, CAM-B3LYP or PBEPBE/ 6-31G(d,p)	Investigation of DES structure	[67]
29	ChCl:Glycerol (1:1)	DFT BLYP/MOLOPT-DZVP-SR-GTH	SO_2_ solvation in DES	[102]
30	ChCl:Glycerol (1:2)	DFT M062x/ 6-31++G(d,p)	Investigation of DES structure	[82]
31	ChCl:Glycerol (1:2) ChCl:Glycerol (1:3) ChCl:Malonic acid (1:1)	DFT B3LYP/6-31+G(d,p)	Mitigation of CO_2_ in DES	[72]
32	ChCl:Imidazole:Ethylene glycol (3:7:14) ChCl:Triazole:Ethylene glycol (3:7:14) ChCl:Tetrazole:Ethylene glycol (3:7:14)	AIMD BLYP triple-ζ valence polarization basis set and GTH pseudopotentials DFT 6-31++G(d,p)	DES for Highly Efficient and Reversible Capture of Ammonia	[412]
33	ChCl:Lactic acid:Ethanol (1:2:1) ChCl:Lactic acid:Ethylene glycol (1:2:1)	Semiempirical method with the PM6 level	Investigation of DES structure	[60]
34	ChCl:Lactic acid (1:1) β-alanine:Lactic acid (1:1)	DFT B3LYP/6-311++G(d,p)	Lidocaine in DES	[55]
35	ChCl:Lactic acid (1:9)	DFT M06-2X functional with the standard 6-311+G(d,p)//6-311G(d,p) basis set.	Lignin dissolution behaviors of DES	[383]
36	ChCl:Levulinic acid (1:2)	DFT B3LYP/6-31+G(d,p)	DES for CO_2_ capture	[52]
37	ChCl:Malonic acid:1, 4-butanediol (1:1:1)	DFT B3LYP/3-21G semiempirical method with the PM6 level	Investigation of DES structure	[247]
38	ChCl:Malonic acid:n-butanol (1:1:1) ChCl:Malonic acid:Iso-butanol (1:1:1) ChCl:Malonic acid:Butandiol (1:1:1)	Semiempirical method with the PM6 level	Investigation of DES structure	[59]
39	ChCl:Oxalic acid ChCl:Citric acid ChCl:Glycerol ChCl:Ethylene glycol Different molar ratios	3–21G basis set	Toxicity assessment and enhanced drug solubility profile of green DES derivatives	[413]
40	ChCl:Phenol (1:2) ChCl:Glycol ethylene (1:2) ChCl:Levulinic acid (1:2)	DFT B3LYP/6-311++G(d,p)	Desulfurization using DES	[71]
41	ChCl:Phenylacetic acid (1:2)	DFT B3LYP/6-31+G(d,p)	CO_2_ absorption with DES	[53]
42	ChCl:R-3-hydroxyl acids Different molar ratios	DFT B3LYP/6-31+G(2d,2p) B3LYP-D3/6-311++G(2d,2p)	Investigation of DES structure	[77]
43	ChCl:Urea (1:1) ChCl:Ethylene glycol (1:1)	AIMD BLYP/triple-ζ double-polarization basis set and Goedecker–Teter–Hutter pseudopotentials	Solvation structure around CO_2_ and SO_2_ in DESs	[90]
44	ChCl:Urea (1:2)	DFT M06e2X/6-311++G(d,p)	Interaction of Cu, Ag and Au nanoparticles with DES	[87]
45	ChCl:Urea (1:2)	DFT B3LYP/6-311++G(d,p)	Investigation of DES structure	[76]
46	ChCl:Urea (1:2)	DFT B3LYP/6-311++G(2d,p)	Investigation of DES structure	[64]
47	ChCl:Urea (1:2)	periodic DFT B3LYP/6-31+G(d,p)	Investigation of DES structure	[70]
48	ChCl:Urea (1:2)	AIMD DFT B3LYP/TZ2P	Water effect on DES structure	[109]
49	ChCl:Urea (1:2) ChCl:Acetylsalicylic acid (1:2) ChCl:Sesamol (1:2) ChCl:Pyrogallol (1:1)	DFT B3LYP/ ug-cc-pVTZ	Solubilization properties and structural characterization in DES	[93]
50	ChCl:Urea (1:2) ChCl:Ethylene glycol (1:2)	DFT M06-2X/6-31++G(d,p)	Extractive Desulfurization of Fuel with DES	[85]
51	ChCl:Urea (1:2) ChCl:Ethylene glycol (1:2) ChCl:Glycerol (1:2)	AIMD DFTB3	Investigation of DES structure	[210]
52	ChCl:Urea (1:2) ChCl:Ethylene glycol (1:2) ChCl:Glycerol (1:2)	DFT B3LYP/cc-pVDZ	Solvatochromic behavior of dimethyl sulfoxide with DESs	[394]
53	ChCl:Urea (1:2) ChCl:Ethylene glycol (1:2) ChCl:Glycerol (1:2) ChCl:Propylene glycol (1:2)	DFT B3LYP/6-311G(d)	Extraction mechanism of 1-butanol separation from alkanol azeotropic system using choline-based DES	[284]
54	ChCl:Urea (1:2) ChCl:Ethylene glycol (1:2) ChCl:Levulinic acid (1:2) Betaine:Levulinic acid (1:2)	AIMD BLYP with a triple-ζ, double polarization basis set for nonmetal atoms, and GTH pseudopotentials	Mercury Capture from Petroleum Using DES	[159]
55	ChCl:Urea (1:2) ChCl:Ethylene glycol (1:2) Choline iodide:Glycerol (1:1) ChCl:Benzoic acid (1:2)	DFT M06-2X/cc-pVDZ level	Adsorption of DESs onto graphene and defective graphene nanoflake	[314]
56	ChCl:Urea (1:2) ChCl:Glycerol (1:2) ChCl:Malonic acid (1:1)	DFT B3LYP/6-311+G(d)	Mechanisms of acid gases capture at relevant interfaces and at atomistic level in DES	[268]
57	ChCl:Urea (1:2) ChCl:Thio urea (1:2) ChCl:Ehylene glycol (1:2) ChCl:Glycerol (1:2)	DFT B3LYP/6-31G	Ammonium-based DES for secondary water flooding	[414]
58	ChCl:Urea (1:2) ChCl:Thio urea (1:2) ChCl:Methyl urea (1:2) ChCl:Dimethyl urea (1:2) ChCl:1,1-dimethylurea (1:2) ChCl:*N*,*N*′-ethylene urea (1:2)	AIMD BLYP-D3(BJ)	Investigation of DES structure	[119]
59	ChCl based DESs HBDs: straight-chain monobasic acids	DFT B3LYP/6-31G(d)	Sulfur removal by DES	[73]
60	ChCl:Urea (1:2)	BLYP-D2/ DZVP-MOLOPT-SR-GTH	Investigation of DES structure	[209]
61	DESs based on LiCl and LiTFSI HBD: Urea, Acetamide, *N*,*N*_0_-dimethylpropyleneurea, 2-imidazolidinone, Tetramethylurea Molar ratio 1:5	DFT B3LIP/6-311++G(d,p)	Investigation of DES structure	[245]
62	L-Arginine:Glutamic acid (1:1) L-Arginine:Oxalic acid (1:1) L-Arginine:Tartaric acid (1:1)	DFT B3LYP/6-31++G(d,p)	Nitric oxide solubility in DES	[152]
63	L-Menthol:Acetic acid (1:1)	ωB97XD/6-311G(d,p)	Structure Elucidation of DES	[155]
64	Lactic acid:Alanine (7:1) Lactic acid:Glycine (7:1) Lactic acid:Histidine (9:1)	DFT B3LYP/6-311G(d,p)	Vapor−liquid equilibria, vapor pressure and water activity for the aqueous solutions of natural deep eutectic solvents (NDESs)	[66]
65	Litium bis-(trifluoromethanesulfonyl)-imide:Urea Different molar ratios	B3LYP/ def2-TZVPP	Investigation of DES structure	[403]
66	Methyltriphenylphosphonium bromide:Ethylene glycol (1:4)	DFT B3LYP/6-31G(d)	Extraction of a polyaromatic hydrocarbon from fuel oils using DES	[283]
67	Methyltriphenylphosphonium bromide:Glycerol (1:2)	HF/6-31G(d)	Adsorption of phenol and crystal violet dye on carbon nanotube functionalized with deep eutectic solvent	[96]
68	Monoethanolamine hydrochloride:Methyldiethanolamine Diethanolamine hydrochloride:Methyldiethanolamine *N*-methyl diethanolamine hydrochloride:Methyldiethanolamine Different molar ratios	DFT B3LYP/6-31G(d,p)	CO_2_ capture performance by DES	[402]
69	Tetrabutylammonium bromide:Formic acid (1:1)	DFT B3LYP/6-311++G(d,p)	Oil desulfuration by DES	[149]
70	Tetrabutylammonium bromide:Imidazole (1:2) Tetrabutylammonium bromide:Ethylene glycol (1:4) Tetrabutylammonium bromide:Glycerol (1:4)	DFT B3LYP/6-311+G(d)	Dissolution of carbohydrates in DES	[338]
71	Tetrabutylphosphonium bromide:Phenol (1:4) Tetrabutylphosphonium bromide:Diethylene glycol (1:4) Allyltriphenylphosphonium bromide:Phenol (1:4) Allyltriphenylphosphonium bromide:Phenol (1:6)	DFT B3LYP/6-31++G(d,p)	CO_2_ solubility in DESs	[278]
72	Tetraethylammonium bromide:Ethylene glycol (1:2) Tetraethylammonium chloride:Ethylene glycol (1:2)	DFT B3LYP/6-31G(d)	Influence of Br^−^ and Cl^−^ on DES	[61]
73	Tetraethylammonium chloride:Lactic acid (1:2) Tetrabutylammonium chloride:Lactic acid (1:2) Benzyltributylammonium chloride:Glycolic acid (1:2) Benzyltributylammonium bromide:Lactic acid (1:2) Dodecyltributylammonium chloride:Lactic acid (1:2) Tetrabutylammonium chloride:Lactic acid (1:2) Benzyltributylammonium chloride:Lactic acid (1:2)	DFT B3LYP/6-31+G(d,p)	DES complex with regulate magnetic (Fe_3_O_4_) metal organic framework	[65]
74	Triethylammonium:Formate Triethylammonium:Acetate Triethylammonium:Propionate Triethylammonium:Butanoate Triethylammonium:Pentanoate Different molar ratios	DFT M06-2X/6-31++G(d,p)	Extractive desulfurization process with DES	[84]

**Table A3:** Machine Learning calculations of DES consisting systems, used method and model, and the topic of the publication.

**No**	**DES**	**Models and Methods**	**Topic of the Publication**	**Reference**
1	DESs based on ammonium salt and on phosphonium salt	Artificial neural network model. A feed forward back propagation neural network with 9 hidden neurons. The group contribution method applied the modified Lydersen–Joback–Reid, Lee–Kesler and the modified Rackett equations	Prediction of DES densities	[186]
2	DESs based on ammonium salt and on phosphonium salt	Artificial neural network model 8-4-1. A feed-forward neural network with 4 hidden neurons Levenberg–Marquardt optimization method	Prediction of glycerol removal from palm-oil based biodiesel using DESs Total glycerol content	[187]
3	DESs based on amine with different HDSs	A combination of multi-linear regression and artificial neural networks methods The stepwise regression algorithm was used for the regression analysis of the experimental viscosity data expressed by s-profile and temperature multi-linear regression model descriptors	Prediction of the viscosity of DESs	[188]
4	DESs based on ChCl	Response surface methodology and Artificial neural networking	Study of the efficacy of 10 NDES (natural DES), including 3 new NDES, to extract procyanidins and anthocyanins from cranberry pomace	[189]
5	DESs based on ChCl	Artificial neural network model. A total of two types of neural network to analyze the feed-forward back-propagation and the layer recurrent	Prediction of lead removal from water using DES functionalized CNTs	[190]
6	*N*,*N*-diethylethanolammonium chloride:Glycerol (2:1)	The non-linear autoregressive network with exogenous inputs neural network strategy. The back-propagation training algorithm was selected to update the bias and weight vector values corresponding to the momentum, and the tangent sigmoid transfer function (tansig) was selected as the neuron transfer function for the network	Prediction of arsenic removal from water solution using DES functionalized CNTs	[191]
7	Benzyltriphenylphosphonium chloride:Glycerol (16:1)	The non-linear autoregressive network with exogenous inputs (NARX) neural network strategy. Three kinetic models were used to identify the adsorption rate and mechanism, and the pseudo-second order best described the adsorption kinetics	Prediction of arsenic removal from water solution using DES functionalized CNTs	[192]
8	Allyl triphenyl phosphonium bromide:Glycerol	Artificial neural network The non-linear autoregressive exogenous model network Feedforward neural network and layer recurrent neural network	Prediction of mercury removal from water solution using DES functionalized CNTs	[193]
9	DESs based on acid ChCl and Levulinic acid as HBAs	Particle Swarm Optimization to optimize an Artificial Neural Network Adaptive-network-based fuzzy inference system and particle swarm optimization Least square support vector Multilayer perceptron	Modeling of CO_2_ solubility in various DESs	[194]
10	DESs based on ChCl, *N*,*N*-diethyl ethanol ammonium chloride, and methyl triphenyl phosphonium bromide salts	Artificial neural network model. Feed-forward back propagation neural network with 8 hidden neurons	Prediction of the electrical conductivity DESs at different temperatures and compositions	[195]
11	Choline chloride:citric acid 1:1 Choline chloride: monohydrate 1:1	Artificial neural network model. Artificial neural network and genetic algorithm approach The feed-forward backpropagation neural network algorithm with 4 input layer neurons and 2 output layer neurons for 4 independent and 2 dependent variables. The optimum number of hidden layer neurons was 11	Experiment design for microwave-assisted extraction of phytochemical compounds from black jamun pulp	[196]
12	DESs based on allyltriphenylphosphonium bromide: Triethylene glycol with molar ratios of 1:4, 1:10 and 1:16	Linear and quadratic regression models	Estimation of carbon dioxide solubility in DESs	[198]
13	DESs based on ChCl: Glycerol ChCl: P-coumaric acid	Principal component analysis Partial least squares Furthermore, based on molecular simulation, the detailed relationships between key variables were further analyzed	Revealing the biomass pretreatment mechanism by evaluating the inner relationships among 42 key process factors	[199]
14	DESs based on tetraalkylammonium bromide	Principal component analysis Regression analysis	Prediction of DES eutectic temperatures	[200]
